# Safety and Immunogenicity of mRNA-1010, an Investigational Seasonal Influenza Vaccine, in Healthy Adults: Final Results From a Phase 1/2 Randomized Trial

**DOI:** 10.1093/infdis/jiae329

**Published:** 2024-06-27

**Authors:** Jintanat Ananworanich, Ivan T Lee, David Ensz, Lizbeth Carmona, Kristi Schaefers, Andrei Avanesov, Daniel Stadlbauer, Angela Choi, Alicia Pucci, Shannon McGrath, Hsiao-Hsuan Kuo, Carole Henry, Ren Chen, Wenmei Huang, Raffael Nachbagauer, Robert Paris

**Affiliations:** Moderna, Inc, Cambridge, Massachusetts; Moderna, Inc, Cambridge, Massachusetts; Meridian Clinical Research, Sioux City, Iowa; Moderna, Inc, Cambridge, Massachusetts; Moderna, Inc, Cambridge, Massachusetts; Moderna, Inc, Cambridge, Massachusetts; Moderna, Inc, Cambridge, Massachusetts; Moderna, Inc, Cambridge, Massachusetts; Moderna, Inc, Cambridge, Massachusetts; Moderna, Inc, Cambridge, Massachusetts; Moderna, Inc, Cambridge, Massachusetts; Moderna, Inc, Cambridge, Massachusetts; Moderna, Inc, Cambridge, Massachusetts; Moderna, Inc, Cambridge, Massachusetts; Moderna, Inc, Cambridge, Massachusetts; Moderna, Inc, Cambridge, Massachusetts

**Keywords:** influenza, messenger RNA, phase 1/2, randomized clinical trial, vaccine

## Abstract

**Background:**

Seasonal influenza remains a global public health concern. A messenger RNA (mRNA)–based quadrivalent seasonal influenza vaccine, mRNA-1010, was investigated in a first-in-human, phase 1/2 clinical trial conducted in 3 parts.

**Methods:**

In parts 1 to 3 of this stratified observer-blind study, adults aged ≥18 years were randomly assigned to receive a single dose (6.25–200 µg) of mRNA-1010 or placebo (part 1) or an active comparator (Afluria; parts 2 and 3). Primary study objectives were assessment of safety, reactogenicity, and humoral immunogenicity of mRNA-1010, placebo (part 1), or active comparator (parts 2 and 3). Exploratory end points included assessment of cellular immunogenicity (part 1) and antigenic breadth against vaccine heterologous strains (A/H3N2; parts 1 and 2).

**Results:**

In all study parts, solicited adverse reactions were reported more frequently for mRNA-1010 than placebo or Afluria, and most were grade 1 or 2 in severity. No vaccine-related serious adverse events or deaths were reported. In parts 1 and 2, a single dose of mRNA-1010 (25–200 µg) elicited robust day 29 hemagglutination inhibition titers that persisted through 6 months. In part 3, lower doses of mRNA-1010 (6.25–25 µg) elicited day 29 hemagglutination inhibition titers that were higher or comparable to those of Afluria for influenza A strains. When compared with Afluria, mRNA-1010 (50 µg) elicited broader A/H3N2 antibody responses (part 2). mRNA-1010 induced greater T-cell responses than placebo at day 8 that were sustained or stronger at day 29 (part 1).

**Conclusions:**

Data support the continued development of mRNA-1010 as a seasonal influenza vaccine.

**Clinical Trials Registration:**

NCT04956575 (https://clinicaltrials.gov/study/NCT04956575).

## Teaser

A single dose of mRNA-1010 quadrivalent seasonal influenza vaccine elicited broad and durable humoral responses that persisted through 6 months, as well as robust T-cell responses, with a safety profile consistent with other mRNA-based vaccines.

Influenza remains a global public health burden, with approximately 1 billion cases worldwide annually [1] . Influenza A and B viruses are responsible for most influenza-associated hospitalizations and deaths and cause seasonal epidemics [1–3]. Although influenza affects all age groups, older adults are more susceptible to influenza-associated complications, with 67% of influenza deaths worldwide occurring in those aged ≥65 years [4].

Vaccination is a crucial public health strategy against influenza; however, effectiveness of available vaccines varies due to poor vaccine uptake, weak or short-lived immunity, and mismatch with circulating strains [1, 5]. Strain mismatch can occur during the lengthy production process required with traditional influenza vaccines, as well as adaptive mutations from virus propagation in egg-based platforms [6–8]. Antigenic drift, a natural feature of influenza viruses, also poses distinct challenges by creating diverse viral subtypes and enabling influenza viruses to continuously circulate in the human population and evade immune detection [6, 9, 10]. While all influenza viruses undergo antigenic drift, the frequency is higher for A/H3N2 vs A/H1N1 and B strains [5, 10, 11]. Accordingly, vaccine effectiveness is typically lower against A/H3N2 [10, 12]. Yet, even when vaccines are well matched to circulating strains, vaccine effectiveness is estimated as 40% to 60% [5].

mRNA-1010 is a quadrivalent seasonal influenza vaccine encoding the hemagglutinin (HA) surface glycoproteins of strains recommended by the World Health Organization: A/H1N1, A/H3N2, B/Victoria, and B/Yamagata [1]. The mRNA platform could improve on limitations of available influenza vaccines, such as avoiding egg-acquired mutations, reducing strain mismatch with shorter manufacturing time frames, and inducing strong humoral and cellular immune responses to increase durability of protection [13, 14]. Interim findings from a phase 1/2 clinical trial in healthy adults showed that a single mRNA-1010 dose had no safety concerns and elicited a robust immune response against vaccine-included strains through 28 days after vaccination [15]. Here, safety and humoral immunogenicity through 6 months after vaccination are presented, as well as antigenic breadth against A/H3N2 viruses and cellular immunogenicity.

## METHODS

### Study Design and Participants

This first-in-human, phase 1/2 study based on a randomized observer-blinded design was conducted at 20 sites in the United States to evaluate mRNA-1010 safety, reactogenicity, and immunogenicity in adults aged ≥18 years (ClinicalTrials.gov: NCT04956575). The study comprised 3 parts, with part 1 assessing mRNA-1010 vs placebo and parts 2 and 3 being a dose-ranging study of mRNA-1010 vs licensed comparator vaccines. Interim findings for parts 1 and 2 were previously reported [15]. This report summarizes results for parts 1 to 3 after all participants had completed or withdrawn from the study.

Eligible participants were healthy adults aged ≥18 years (part 1) or medically stable adults aged ≥18 years, excluding those with chronic diseases requiring ongoing medical intervention within the 3 months before enrollment and with immunocompromising conditions or medications (parts 2 and 3). Participants were excluded from the study if they had received a seasonal influenza vaccine or any other investigational influenza vaccine after 1 January 2021 (part 1), within 6 months prior to the screening visit (part 2), or within 180 days prior to randomization (part 3). Inclusion/exclusion criteria are provided in the Supplementary Material.

The study design has been described previously (Supplementary Figure 1) [15]. Briefly, 180 participants in part 1 were randomly assigned (1:1:1:1) to receive a single dose of mRNA-1010 (50, 100, or 200 µg) or placebo. In part 2, 503 participants were randomly assigned (3:3:3:1) to receive a single dose of mRNA-1010 (25, 50, or 100 µg) or licensed quadrivalent seasonal influenza vaccine (Afluria; Seqirus Pty Ltd). In part 3, 202 participants were randomly assigned (1:1:1:1) to receive a single dose of mRNA-1010 (6.25, 12.5, or 25 µg) or licensed quadrivalent influenza vaccine (Afluria). Details on randomization and blinding are presented in the Supplementary Material. Participants received a single dose of the vaccine on day 1, and the final visit was on day 181 (month 6).

The study was conducted in accordance with the protocol, applicable laws, and regulatory requirements; the good clinical practice guidelines of the International Council for Harmonization; and the consensus ethical principles derived from international guidelines, including the Declaration of Helsinki and Council for International Organizations of Medical Sciences. The protocol was approved by the central institutional review board (Advarra, Inc) prior to study initiation, and written informed consent was obtained from all participants before enrollment.

### Vaccines

mRNA-1010 comprises mRNAs encoding surface glycoprotein HA of 4 influenza virus strains formulated in lipid nanoparticles. Strain selection for mRNA-1010 was based on World Health Organization recommendations for the 2021 Southern Hemisphere vaccine composition (part 1) and the 2021/2022 Northern Hemisphere vaccine composition (parts 2 and 3; Supplementary Material). mRNA-1010 was provided as a sterile liquid for injection and diluted to different dose levels with normal saline. Placebo (part 1) consisted of normal saline; active comparator (parts 2 and 3) consisted of licensed quadrivalent influenza vaccine (Afluria). All vaccines were administered intramuscularly as a single 0.5-mL injection.

### Objectives

Part 1 primary objectives were to evaluate the safety and reactogenicity of mRNA-1010 vs placebo and to evaluate the humoral immunogenicity of mRNA-1010 against vaccine-matched influenza A and B strains at day 29. Secondary objectives were to evaluate the humoral immunogenicity against vaccine-matched strains at all evaluable time points (days 8, 29, and 181). Exploratory end points included evaluation of the cellular immunogenicity (days 8 and 29) and humoral immunogenicity against vaccine-mismatched strains in a subset of participants.

The primary and secondary objectives of parts 2 and 3 were to evaluate mRNA-1010 vs licensed influenza vaccine for safety, reactogenicity, and humoral immunogenicity against vaccine-matched influenza A and B strains at day 29. Exploratory objectives included evaluations of humoral immunogenicity against vaccine-matched or vaccine-mismatched strains (part 2) and humoral immunogenicity in a subset of participants at days 91 and 181 (parts 2 and 3).

### Safety Assessments

Safety end points included solicited local and systemic adverse reactions (ARs) for 7 days after vaccination and unsolicited adverse events (AEs) for 28 days after vaccination, as well as serious AEs, AEs of special interest, and medically attended AEs throughout the study (day 181).

### Immunogenicity Assessments

Blood samples for immunogenicity assessments were collected on days 1 (baseline), 8 (part 1 only), 29, 91 (parts 2 and 3 only), and 181 (end of study; humoral immunogenicity only). Humoral immunogenicity end points included geometric mean titers (GMTs) at days 1, 8, 29, and 181; geometric mean fold rises (GMFRs) at days 8, 29, and 181 over day 1; percentage of participants at day 29 with seroconversion of serum anti-HA antibodies as measured by hemagglutination inhibition (HAI) assay [15] (Supplementary Material); and percentage of participants with HAI titers ≥1:40 at day 29, 91, or 181. Antigenic breadth end points consisted of GMTs and GMFRs at day 29 for vaccine-heterologous A/H3N2 strains as measured by HAI assay. Cellular immunogenicity end points comprised the frequency and magnitude of HA-specific CD4+ and CD8+ nonnaive T-cell responses measured by ex vivo stimulation and intracellular staining assay (Supplementary Material).

### Statistical Analyses

No formal statistical hypotheses were tested. Sample size (Supplementary Material) was considered sufficient to provide a descriptive summary of the safety and immunogenicity of different dose levels of mRNA-1010. All safety assessments except for solicited local and systemic ARs were assessed in the safety population, which included all randomized participants who received vaccination. Solicited ARs were assessed in all participants in the safety population who contributed any solicited AR data (solicited safety population). Participants used an electronic diary to record local ARs (ie, injection site pain, redness, or hardness or axillary swelling/tenderness ipsilateral to the side of injection) or systemic ARs (ie, headache, fatigue, myalgia, arthralgia, nausea/vomiting, chills, and fever). The number of events of unsolicited AEs, serious AEs, AEs of special interest, and medically attended AEs were summarized, while descriptive summary statistics were provided for all other safety analyses.

Immunogenicity analyses were performed in the per-protocol population, which included all randomly assigned participants who received vaccination and complied with baseline and ≥1 postvaccination time point blood sampling, did not have influenza infection at baseline through day 29 (as documented by polymerase chain reaction), and had no major protocol deviation that affected the immune response. GMTs with corresponding 95% CIs and GMFRs over day 1 (baseline) with corresponding 95% CIs were calculated for each vaccination group; 95% CIs were calculated per the *t* distribution of the log_2_-transformed values and then back-transformed to the original scale. The seroconversion rate from baseline was determined along with 2-sided 95% CIs per the Clopper-Pearson method. The rate of seroconversion was defined as the percentage of participants with either a prevaccination HAI titer <1:10 and a postvaccination HAI titer ≥1:40 or a prevaccination HAI titer ≥1:10 and a minimum 4-fold rise in postvaccination HAI antibody titer. In parts 2 and 3, GMTs and seroconversion rates at day 29 were compared in the mRNA-1010 groups with the active comparator group. Descriptive summary statistics were provided for virus-specific T-cell responses. Statistical analyses were performed with SAS version 9.4 (SAS Institute). Antibody titers reported as less than the lower limit of quantitation were replaced by 0.5× lower limit of quantitation; antibody titers reported as greater than the upper limit of quantitation were converted to the upper limit of quantitation.

## RESULTS

### Participants

**Figure 1.**
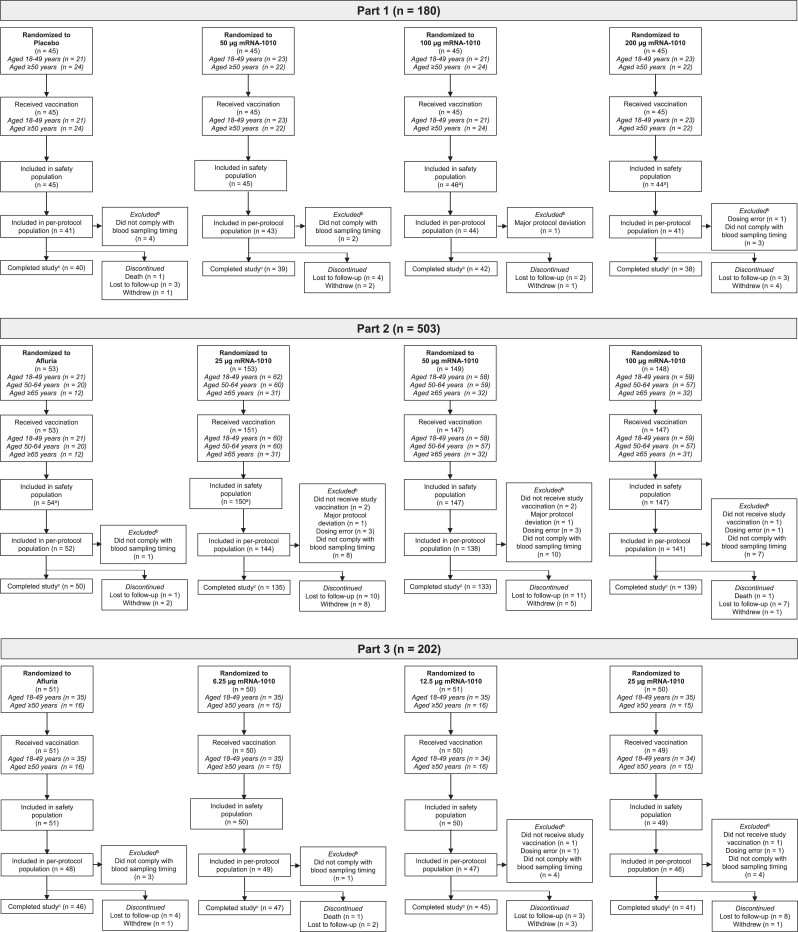
Participant disposition by study part. All randomly assigned participants who received study vaccination were included in the safety population; participants were included in the group based on the actual vaccine received. The immunogenicity per-protocol population included all randomly assigned participants who received vaccination and complied with baseline and ≥1 postvaccination time point blood sampling, did not have influenza infection at baseline through day 29 (as documented by polymerase chain reaction), and had no major protocol deviation that affected the immune response. ^a^ There was 1 dosing error in part 1 (1 participant randomly assigned to mRNA-1010 at 200 µg but received 100 µg) and 2 dosing errors in part 2 (1 participant randomly assigned to mRNA-1010 at 25 µg but received 50 µg; 1 participant randomly assigned to mRNA-1010 at 50 µg but received active comparator). ^b^ Participants could contribute to multiple reasons for exclusion from the immunogenicity per-protocol population. ^c^ Participants were considered to have completed the study if they completed the final study visit at day 181. mRNA, messenger RNA.

The first participant was randomly assigned on 6 July 2021, and the study was completed on 25 October 2022; the database lock date was 5 December 2022. Overall, 736 participants were randomly assigned to mRNA-1010 groups, with 45 to placebo (part 1) and 104 to Afluria (parts 2 and 3; Figure 1). Most participants (90%, 795/885) completed the study; reasons for study discontinuation were balanced among groups.

Most participants were female, White, and non-Hispanic or Latino (Supplementary Table 1). The median ± SD age of participants was lower in part 3 (40.7 ± 15.2 years) as compared with parts 1 (49.8 ± 15.9 years) and 2 (50.2 ± 16.0 years). Overall, 60.1% of mRNA-1010 recipients had not received any seasonal influenza vaccine in the previous season at the time of randomization. All other baseline characteristics were comparable among vaccination groups.

### Safety

For all 3 study parts combined, rates of any solicited local and systemic ARs within 7 days were higher after mRNA-1010 than after placebo or Afluria vaccination (Supplementary Figure 2). A dose response was observed for mRNA-1010, and more solicited ARs were reported for younger participants (18–49 years) than older ones (≥50 years). No grade 4 solicited ARs were reported in any group. The most common solicited local AR across vaccine groups was injection site pain (Supplementary Figure 3), which was predominantly grade 1, except for the 200-µg mRNA-1010 group (predominantly grade 2). Fatigue, myalgia, headache, and arthralgia were the most common solicited systemic ARs, which were predominantly grade 1 or 2 (Supplementary Figure 3).

Within mRNA-1010 groups, unsolicited treatment-emergent AEs related to vaccination were reported in 6.2% to 20.6% of participants aged 18 to 49 years and 0% to 10.5% of participants aged ≥50 years within 28 days of vaccination vs a respective 9.5% and 0% of placebo recipients and 7.1% and 6.1% of Afluria recipients (Supplementary Table 2). There were no related serious AEs, AEs of special interest, or treatment-emergent AEs leading to study discontinuation or deaths in any group. Severe related treatment-emergent AEs occurred in 9 mRNA-1010 recipients: fatigue (n = 5), headache (n = 4), injection site erythema (n = 1), injection site lymphadenopathy (n = 1), and injection site pain (n = 1). Medically attended AEs related to vaccination occurred in 4 mRNA-1010 recipients: upper respiratory tract infection (n = 1), ecchymosis (n = 1), injection site pain (n = 1), and injection site lymphadenopathy (n = 1). Three deaths were reported during the study, all of which the investigator considered unrelated to study vaccination (Supplementary Material).

### Immunogenicity

#### Humoral Immune Responses

In part 1, mRNA-1010 (50, 100, and 200 µg) elicited HAI titers against all vaccine-matched influenza strains 28 days after vaccination (day 29) in participants aged 18 to 49 and ≥50 years. HAI titers gradually declined through day 181, but A strain titers remained above baseline for both age groups. Immune responses after placebo remained close to baseline values (Supplementary Table 3).

**Figure 2.**
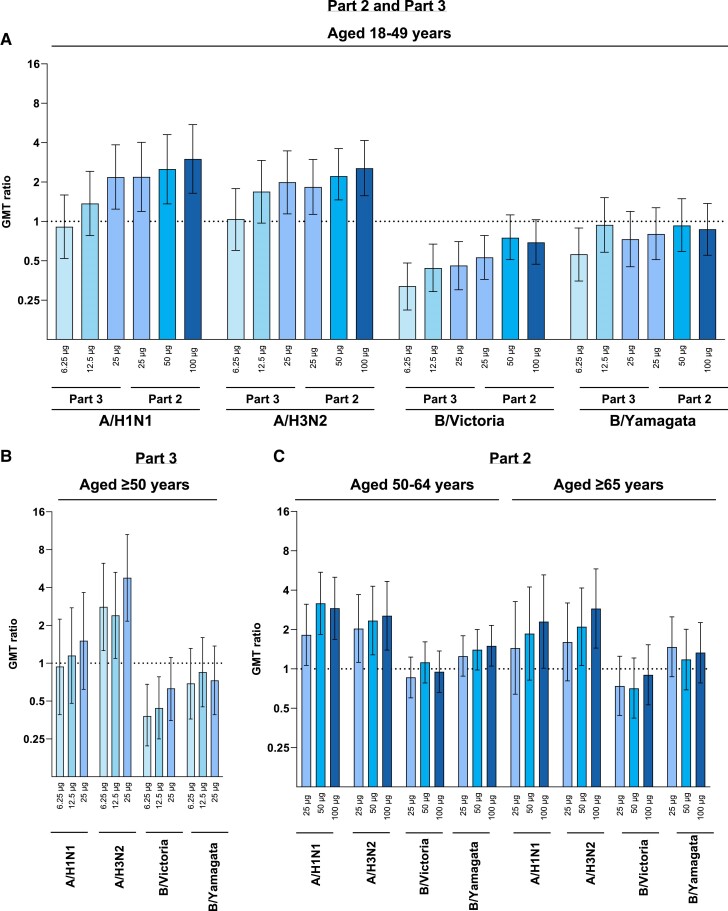
Ratios of GMTs of antihemagglutinin antibodies after vaccination with mRNA-1010 as compared with Afluria at day 29 for parts 2 and 3. Ratios of hemagglutination inhibition GMTs with associated 95% CIs against vaccine-matched seasonal influenza strains at day 29 after vaccination with mRNA-1010 as compared with Afluria in participants aged (*A*) 18 to 49 years in parts 2 and 3, (*B*) ≥50 years in part 3, and (*C*) 50 to 64 years and ≥65 years in part 2. Horizontal dotted line indicates a GMT ratio of 1, which shows comparable GMTs between mRNA-1010 and Afluria. Numbers of participants in the Afluria groups were as follows: 20 (18–49 years), 20 (50–64 years), and 12 (≥65 years) in part 2 and 32 (18–49 years) and 16 (≥50 years) in part 3. Numbers of participants aged 18 to 49 years in the mRNA-1010 groups were as follows: 34 (6.25 µg), 32 (12.5 µg), 31 (25 µg; part 3), 55 (25 µg; part 2), 53 (50 µg), and 57 (100 µg). In part 2, the numbers of participants in mRNA-1010 groups aged 50 to 64 years were 58 (25 µg), 54 (50 µg), and 54 (100 µg), and the numbers of those aged ≥65 years were 31 (25 µg), 31 (50 µg), and 30 (100 µg). In part 3, the numbers of participants aged ≥50 years in the mRNA-1010 groups were 15 (6.25 µg), 15 (12.5 µg), and 15 (25 µg). The numbers of participants are from the per-protocol population. Lower limits of quantitation were 10 for each influenza strain; upper limits of quantitation were 6400 (H1N1 and B/Yamagata), 1280 (H3N2), and 3200 (B/Victoria). GMT, geometric mean titer; mRNA, messenger RNA.

**Figure 3.**
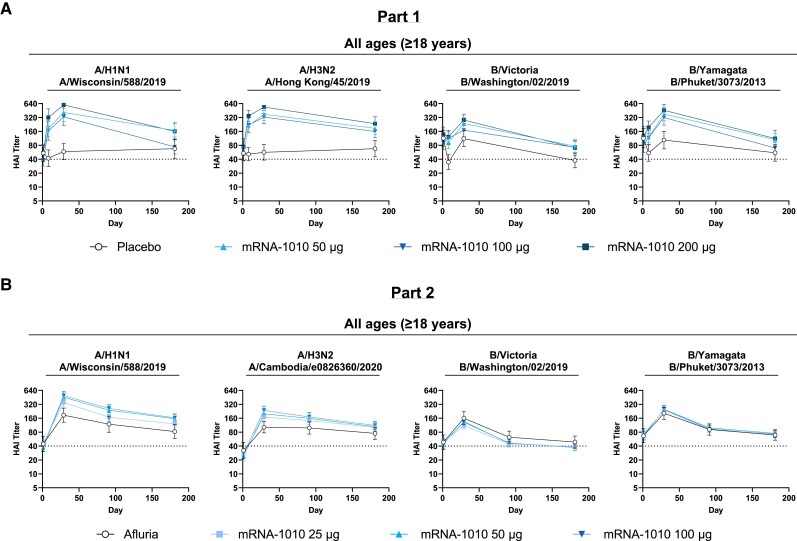
Persistence of antihemagglutinin antibodies after mRNA-1010 vaccination for all ages (≥18 years) in parts 1 and 2. Geometric mean titers with associated 95% CIs against vaccine-matched seasonal influenza strains at (*A*) days 1 (baseline), 8, 29, and 181 across all age groups combined for part 1 and (*B*) days 1 (baseline), 29, 91, and 181 across all age groups combined for part 2. Horizontal dotted line indicates 1:40 titer associated with a 50% reduction in risk of infection [16]. In part 1, the number of participants in the placebo group was 41. The numbers of participants in the mRNA-1010 groups were 43 (50 µg), 44 (100 µg), and 41 (200 µg). In part 2, the number of participants in the Afluria group was 52. The numbers of participants in mRNA-1010 groups were 144 (25 µg), 138 (50 µg), and 141 (100 µg). Numbers of participants were derived from the per-protocol population for both study parts. Days 1 and 29 titers were simultaneously tested, with days 8 and 181 titers tested noncontemporaneously. HAI, hemagglutination inhibition; mRNA, messenger RNA.

In part 2, HAI GMTs at day 29 for mRNA-1010 (25, 50, and 100 µg) were generally higher in younger participants (18–49 years) than older ones (≥50 years) for A/H1N1, A/H3N2, and B/Yamagata at the corresponding dose levels; GMTs were similar between age groups for B/Victoria. For A strains, all mRNA-1010 groups elicited GMTs at day 29 regardless of age group (18–49, 50–64, and ≥65 years) that generally exceeded GMTs elicited by Afluria (Figure 2, Supplementary Table 4). For B strains, GMTs at day 29 for all mRNA-1010 groups (25, 50, and 100 µg) were similar to Afluria (Supplementary Table 4). At day 181, GMTs for A strains were generally higher for all mRNA-1010 groups than Afluria and remained above the 1:40 threshold associated with a 50% reduced risk of infection [16] (Figure 3). GMTs for B strains decreased to baseline levels by day 181 for all mRNA-1010 groups but generally remained at or above the 1:40 threshold [16] (Supplementary Table 4; Figure 3). HAI GMTs by age group through day 181 are shown in Supplementary Figure 4.

In part 3, GMTs elicited by mRNA-1010 (6.25, 12.5, 25 µg) at day 29 were generally consistent across age groups (18–49 and ≥50 years) for A and B strains, except for mRNA-1010 at 12.5 µg, which induced lower GMTs for B/Yamagata in older adults than younger adults (Supplementary Table 5). GMTs elicited by mRNA-1010 at 12.5 and 25 μg were generally higher for A strains than those induced by Afluria, whereas GMTs were similar between mRNA-1010 at 6.25 μg and Afluria. For B strains, GMTs induced by mRNA-1010 were lower than those elicited by Afluria (Figure 2).

#### Antigenic Breadth

**Figure 4.**
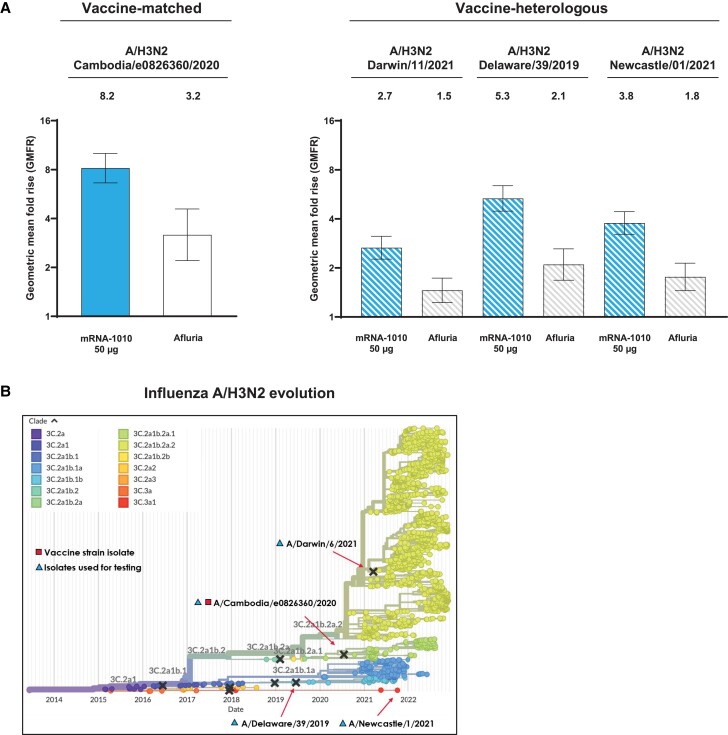
Antigenic breadth of mRNA-1010 against vaccine-heterologous A/H3N2 strains at day 29 in part 2. *A*, Geometric mean fold rise of titers from baseline to day 29 with associated 95% CI among mRNA-1010 (50 µg) and Afluria recipients against vaccine-matched H3N2 (A/Cambodia/e0826360/2020) and vaccine-heterologous H3N2 strains (A/Newcastle/01/2021, A/Delaware/39/2019, and A/Darwin/11/2021). Numbers of participants for the vaccine-matched strain were 52 (Afluria) and 138 (mRNA-1010, 50 µg); for vaccine-heterologous strains, 52 (Afluria) and 139 (mRNA-1010, 50 µg). Numbers of participants for the vaccine-matched strain and vaccine-heterologous strains were derived from the per-protocol population for part 2 of the study. *B*, Phylogenetic tree demonstrating real-time tracking of A/H3N2 clades (adapted from Nextstrain.org) [27].

In a subset of part 2 participants, vaccination with mRNA-1010 at 50 µg induced higher HAI titers (Supplementary Figure 5) against vaccine-heterologous A/H3N2 strains at day 29 when compared with Afluria. Day 29 GMFRs from baseline were 2.7, 5.3, and 3.8 (mRNA-1010, 50 µg) vs 1.5, 2.1, and 1.8 (Afluria) for A/Darwin/11/2021, A/Delaware/39/2019, and A/Newcastle/01/2021, respectively (Figure 4). Antigenic breadth in part 1 is shown in Supplementary Figure 6.

#### Cellular Immune Responses

**Figure 5.**
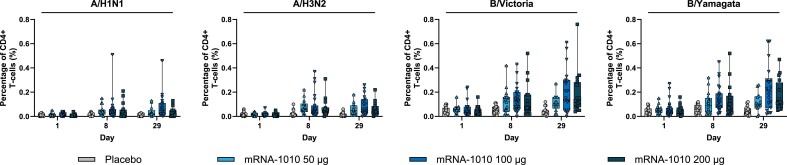
Frequency of type 1 polyfunctional CD4+ T cells after ex vivo stimulation with vaccine strain–specific peptide pools among recipients receiving mRNA-1010 and placebo in part 1. Three type 1 cytokines (IFN-γ, IL-2, and TNF-α) and CD40L functional markers were measured to assess T-cell response. Populations of CD4+ T cells coexpressing all 3 type 1 cytokines and CD40L functional markers from baseline to day 29 are presented. Numbers of participants in the placebo and mRNA-1010 groups were as follows: 23 (placebo), 19 (50 µg), 28 (100 µg), and 24 (200 µg). Boxes represent the 25th and 75th percentiles; whiskers represent the minimum and maximum values. Symbols represent individual-level data. IFN-γ, interferon γ; IL-2, interleukin 2; TNF-α, tumor necrosis factor α.

In part 1, preexisting influenza strain-specific CD4+ T-cell responses were observed at baseline across the mRNA-1010 (50, 100, and 200 µg) and placebo groups, indicating previous influenza exposure (Figure 5, Supplementary Figure 7). When compared with placebo, higher frequencies of influenza strain–specific polyfunctional CD4+ T-cells (simultaneously expressing interferon γ [IFN-γ], interleukin 2, tumor necrosis factor α, and CD40L) were observed at days 8 and 29 for all mRNA-1010 groups (Figure 5). Frequencies of IFN-γ–producing CD4+ T cells (minimally coexpressing IFN-γ and CD40L), which are representative of the Th1 cytokine response [17], increased in an apparent dose-dependent manner among mRNA-1010 groups for B strains and were higher than placebo for all dose groups at days 8 and 29. IFN-γ responses to influenza A strains were lower than responses to B strains; however, higher frequencies of IFN-γ–producing CD4+ T cells were observed at days 8 and 29 for all mRNA-1010 groups as compared with placebo (Supplementary Figure 7). Across all mRNA-1010 groups, CD4+ T-cell responses at day 29 were generally sustained or stronger than those detected at day 8. mRNA-1010 vaccine–induced CD8+/IFN-γ+ T-cell responses were observed for influenza B strains but were not substantially different from placebo (Supplementary Figure 8).

## DISCUSSION

This article presents findings from parts 1 to 3 of a phase 1/2, first-in-human clinical study assessing the safety and immunogenicity of an investigational mRNA-based quadrivalent vaccine (mRNA-1010) against seasonal influenza through 6 months after vaccination in adults aged ≥18 years. mRNA-1010 demonstrated durable humoral responses, higher influenza A HAI GMTs and broader antibody responses than a licensed quadrivalent influenza vaccine (Afluria), and robust cellular CD4+ T-cell responses as compared with placebo. These findings suggest that mRNA-1010 is a promising seasonal influenza vaccine candidate and support its continued clinical development.

A dose-dependent reactogenicity profile was observed for mRNA-1010, with better tolerability at dose levels <100 µg. Incidences of solicited ARs were higher after mRNA-1010 than placebo or Afluria, but most were grade 1 or 2 in severity. No treatment-related serious AEs were reported, and no safety concerns were identified. Overall, the safety profile of mRNA-1010 is consistent with other mRNA-based vaccines [18–21].

The durability of mRNA-1010 vaccination to maintain immune responses throughout an influenza season, which lasts approximately 7 months, was examined. A single dose of mRNA-1010 in part 1 or 2 induced robust day 29 antibody responses that declined through 6 months but remained above (A strains) baseline levels. mRNA-1010 elicited immune responses that exceeded (A strains) or were similar (B strains) to titers elicited by Afluria through 6 months regardless of participant age, remaining above the 1:40 threshold associated with a 50% reduced risk of infection [16]. Overall, these findings demonstrate that mRNA-1010 induces persistent immune responses through 6 months.

Low doses of mRNA-1010 were evaluated in part 3 (6.25, 12.5, and 25 µg), eliciting day 29 titers that were generally higher (12.5 and 25 µg) or comparable (6.25 µg) to Afluria for A strains but lower for B strains, with responses generally consistent across younger adults (18–49 years) and older ones (≥50 years). Notably, lower doses of mRNA-1010 (6.25 and 12.5 µg) had comparable or higher titers for A strains than Afluria, indicating that these dose levels may be suitable for pediatric and young adult populations with further vaccine optimization to improve responses to B strains [21]. A phase 3 study assessing the safety and immunogenicity of an updated formulation of mRNA-1010 optimized for improved responses to B strains is ongoing in approximately 8400 adults aged ≥18 years (NCT05827978), with preliminary findings showing improved immunogenicity against B strains [22].

The ability of mRNA-1010 to induce antibody responses to heterologous A/H3N2 strains was also evaluated. Currently, licensed seasonal influenza vaccines primarily rely on eliciting strain-specific antibodies against influenza's HA surface glycoprotein [23–26], with effectiveness largely dependent on vaccines being well matched to circulating strains [25, 26]. However, frequent antigenic drift among A/H3N2 strains across and within seasons can cause low vaccine effectiveness when A/H3N2 seasons predominate [5, 10, 12]. Here, mRNA-1010 (50 µg) elicited broader A/H3N2 antibody responses than Afluria in a subset of participants, as indicated by day 29 GMFRs against several vaccine-heterologous A/H3N2 strains, suggesting that mRNA-1010 can provide broad coverage against distinct A/H3N2 clades that are divergent from the vaccine-included strain.

Data from vaccinated adults aged ≥60 years suggest that T-cell responses may be a better correlate of protection against influenza than humoral immunity in older adults; however, most available influenza vaccines elicit poor T-cell responses [25, 28]. The mRNA platform has been shown to induce strong cellular immune responses and durable germinal center reactions after SARS-CoV-2 vaccination [29, 30], which can increase protection in older adults. Accordingly, this study evaluated T-cell responses after mRNA-1010 vaccination in part 1 participants to ascertain the capability of mRNA-1010 to expand HA-specific responses. CD4+ strain–specific T-cell responses were detected at day 8 and were sustained or stronger at day 29 for all mRNA-1010 groups, with the greatest frequency observed for influenza B strains. Polyfunctional CD4+ T cells (coexpressing 3 type 1 cytokines and CD40L) substantially increased in magnitude after mRNA-1010 vaccination, with IFN-γ–producing CD4+ T cells increasing in frequency after vaccination. While CD8+ T-cell responses, particularly against influenza B strains, were detected at great magnitudes in certain mRNA-1010 recipients, vaccination overall did not induce consistent responses that were substantially different from placebo, due in part to variability observed across participants within vaccine groups. Collectively, these data suggest that mRNA-1010 may overcome limitations of current influenza vaccines by inducing broad and durable humoral immunity as well as T-cell responses, particularly in older adults. Humoral and T-cell immune responses of mRNA-1010 and future mRNA vaccine iterations, as compared with a licensed enhanced seasonal influenza vaccine, are being investigated in a phase 1/2 trial in approximately 600 adults (NCT05333289).

This study was strengthened by the randomized, observer-blind, placebo-controlled (part 1), and active-controlled (part 2–3) design, as well as its multiple assessments on vaccine-mediated immunogenicity. Limitations include the relatively small number of participants enrolled, which restricted statistical comparisons across dose levels, as well as the enrollment of higher percentages of White and non-Hispanic/Latino participants, which may limit generalizability to the broader population. Other phase 3 trials are further evaluating mRNA-1010, including the safety and immunogenicity in approximately 6000 adults aged ≥18 years (NCT05415462) and the safety and efficacy in approximately 23 000 adults aged ≥50 years (NCT05566639).

In conclusion, findings from this phase 1/2 study demonstrated that mRNA-1010 elicited broad and durable humoral responses that persisted through 6 months, as well as robust T-cell responses. Data support the continued development of mRNA-1010 as a seasonal influenza vaccine.

## Supplementary Data

Supplementary materials are available at *The Journal of Infectious Diseases* online (http://jid.oxfordjournals.org/). Supplementary materials consist of data provided by the author that are published to benefit the reader. The posted materials are not copyedited. The contents of all supplementary data are the sole responsibility of the authors. Questions or messages regarding errors should be addressed to the author.

## Supplementary Material

jiae329_Supplementary_Data

## Notes

***Acknowledgments.*** Medical writing and editorial assistance were provided by Ashlea Inan, PhD, and Andy Kerr, PhD, of MEDiSTRAVA in accordance with good publication practice guidelines (GPP 2022), funded by Moderna, Inc, and under the direction of the authors.

***Author contributions.*** J. A., I. T. L., D. E., L. C., K. S., D. S., S. M., R. C., W. H., R. N., and R. P. contributed to the study concept and design. Data were collected by I. T. L., D. E., L. C., A. A., D. S., A. C., A. P., S. M., H.-H. K., C. H., W. H., and R. P. and then analyzed and interpreted by J. A., I. T. L., D. E., L. C., K. S., A. A., D. S., A. C., A. P., S. M., H.-H. K., C. H., R. C., W. H., R. N., and R. P. All authors contributed to the drafting and critical review of this manuscript and approved the final draft.

***Data-sharing statement.*** Access to patient-level data presented in this article (antibody assays, safety, and reactogenicity) and supporting clinical documents with external researchers who provide methodologically sound scientific proposals will be available upon reasonable request and subject to review from 2 years after study completion. Such requests can be made to Moderna Inc, 325 Binney Street, Cambridge, MA 02142. A materials transfer and/or data access agreement with the sponsor will be required for accessing shared data. All other relevant data are presented in the article (ClinicalTrials.gov: NCT04956575).

***Financial support*.** This study was funded by Moderna.
