# Supplementary material for: Safety and Immunogenicity of mRNA-1010, an Investigational Seasonal Influenza Vaccine, in Healthy Adults: Final Results From a Phase 1/2 Randomized Trial
Source: J Infect Dis. 2024 Jun 27;231(1):e113–22. doi: 10.1093/infdis/jiae329 (PMC11793046; doi:10.1093/infdis/jiae329)
Supplement: jiae329_Supplementary_Data [file jiae329_supplementary_data.docx]

**Supplementary Methods**

**Study Design**

In Part 1, after randomization and blinded review of safety in an initial group of 36 participants (9 participants in each group), randomization of remaining participants was age-stratified (18-49 and ≥50 years) and balanced within each group. Randomization was performed in parallel among the 4 vaccine groups in Parts 2 and 3. In Part 2, participants were age-stratified (18-49, 50-64, or ≥65 years) and vaccination status in the previous influenza season (received or not received); in Part 3, participants were age-stratified (18-49 or ≥50 years). The randomized allocation schedule in each part was generated by the sponsor’s biostatistics department or designee.

This was an observer-blind study, with the Investigator, study staff, study participants, site monitors, and Sponsor personnel blinded to the vaccine administered until the study database was locked and unblinded. A limited number of unblinded personnel were assigned to vaccine accountability procedures and prepared the study vaccines, while an unblinded medically qualified study site personnel administered vaccination. Unblinded site monitors were assigned responsibilities to ensure sites were following all proper vaccination accountability, preparation, and administration procedures.

**Inclusion and Exclusion Criteria for Part 1**

Each participant was required to have met the following criteria for enrollment into Part 1 of the study:

- An adult, ≥18 years of age at the time of consent, who was in good health, in the opinion of the Investigator, based on review of medical history and physical examination performed at screening
- Understood and was willing and physically able to comply with protocol-mandated follow-up, including all procedures, in the opinion of the Investigator
- Provided written informed consent for participation in this study, including all evaluations and procedures as specified in the protocol
- Had a body mass index of 18 kg/m^2^ to 35 kg/m^2^ (inclusive) at screening
- If female and not of childbearing potential, could be enrolled in the study. A follicle-stimulating hormone (FSH) level may be measured at the discretion of the Investigator to confirm postmenopausal status.
- If female and of childbearing potential, met all the following criteria to be enrolled in the study:
  - Had a negative pregnancy test at screening and on the day of vaccination (Day 1)
  - Had practiced adequate contraception or abstained from all activities that could result in pregnancy for at least 28 days prior to Day 1. Adequate female contraception was defined as consistent and correct use of a US Food and Drug Administration (FDA)-approved contraceptive method in accordance with the product label
  - Had agreed to continue adequate contraception through 3 months following vaccine administration
  - Was not currently breastfeeding

Participants who met any of the following criteria were excluded from Part 1 of the study:

- Had significant exposure to someone with laboratory-confirmed severe acute respiratory syndrome coronavirus 2 (SARS-CoV-2) infection, coronavirus disease 2019 (COVID-19), or influenza-like illness (ILI) in the past 14 days prior to screening, as defined by the Centers for Disease Control and Prevention (CDC) as close contact with someone who has COVID-19
- Had a positive SARS-CoV-2 reverse transcription-polymerase chain reaction (RT-PCR) or antigen test in the 10 days prior to screening
- A participant who had a positive SARS-CoV-2 serological test in the 10 days prior to screening but was otherwise asymptomatic and did not have a positive SARS-CoV-2 RT-PCR or antigen test at screening could still be enrolled if they met the study eligibility criteria
- A participant who had a positive SARS-CoV-2 RT-PCR or antigen test in the 10 days prior to screening could be enrolled 10 days after symptom onset or 10 days after the date of the first positive SARS-CoV-2 RT-PCR test without repeat testing, provided the participant was asymptomatic and met the study eligibility criteria, as well as the CDC recommendation for duration of isolation and precautions
- Additionally, a participant who met all study eligibility criteria (note fourth exclusion criterion bullet) at screening but later tested positive for SARS-CoV-2 at Visit 1 would continue participation in the study
- Had clinical screening laboratory values (white blood cell count, hemoglobin, platelets, alanine aminotransferase, aspartate aminotransferase, creatinine, alkaline phosphatase, and total bilirubin) > Grade 1
- Was acutely ill or febrile (body temperature ≥38.0°C/100.4°F) 72 hours prior to or at screening or Day 1. Participants meeting this criterion could be rescheduled within the 28-day screening window and would retain their initially assigned participant number
- Had a preexisting medical condition that was not stable, at the discretion of the Investigator. A stable medical condition was defined as disease not requiring significant change in therapy or hospitalization for worsening disease during the 2 months before enrollment. Participants could be rescreened if they were not medically stable at screening. Medical conditions included (but were not limited to) the following:
- Uncontrolled hypertension or systolic blood pressure >150 mm Hg or diastolic blood pressure >90 mm Hg at screening
- Congestive heart failure
- Unstable angina or exacerbation of coronary artery disease within 6 months before the day of vaccination (Day 1) that required cardiac intervention or new cardiac medications to control symptoms
- Diabetes requiring the use of medicine (injectable or oral) or not controlled with diet
- Chronic obstructive pulmonary disease, asthma requiring daily use of a bronchodilator or inhaled/systemic corticosteroids, or other chronic lung diseases such as pulmonary fibrosis
- Had a medical, psychiatric, or occupational condition that could pose additional risk as a result of participation or that could interfere with safety assessments or interpretation of results according to the Investigator’s judgment
- Had a current or previous diagnosis of immunocompromising condition, immune-mediated disease, or other immunosuppressive condition
- Had received systemic immunosuppressants or immune-modifying drugs for >14 days in total within 6 months prior to screening (for corticosteroids ≥10 mg/day of prednisone or equivalent) or was anticipating the need for immunosuppressive treatment at any time during participation in the study
- Had a history of anaphylaxis, urticaria, or other significant adverse reaction requiring medical intervention after receipt of a vaccine or any of the components contained in the investigational vaccine
- Had a history of coagulopathy or bleeding disorder considered a contraindication to intramuscular injection or phlebotomy
- Had received or planned to receive any licensed vaccine ≤28 days prior to the vaccination (Day 1) or planned to receive a licensed vaccine within 28 days after vaccination, with the exception of vaccines authorized or approved for the prevention of COVID-19 (regardless of type of vaccine) that become available to participants during the study. Efforts were made to space study vaccination and COVID-19 vaccination by at least 7 and preferably 14 days, but COVID-19 vaccination should not have been delayed
- Had received a seasonal influenza vaccine or any other investigational influenza vaccine after January 1, 2021
- Had received systemic immunoglobulins or blood products within 3 months prior to screening or had plans to receive them during the study
- Had a diagnosis of malignancy within the previous 10 years (excluding nonmelanoma skin cancer)
- Had donated ≥450 mL of blood products within 28 days prior to screening or planned to donate blood products during the study
- Had participated in an interventional clinical study within 28 days prior to screening based on the medical history interview or planned to do so while participating in this study
- Was an immediate family member or household member of study personnel, study site staff, or Sponsor personnel

**Inclusion and Exclusion Criteria for Parts 2 and 3**

Each participant was required to have met the following criteria for enrollment into Parts 2 or 3 of the study:

- An adult, ≥18 years of age at the time of consent, who in the opinion of the Investigator, was medically stable based on review of medical history and physical examination performed at screening. Medically stable was defined as disease not requiring significant change in therapy or hospitalization for worsening disease during the 3 months before enrollment. Participants could be rescreened if they were not medically stable at screening
- Understood and was willing and physically able to comply with protocol-mandated follow-up, including all procedures, in the opinion of the Investigator
- Provided written informed consent for participation in this study, including all evaluations and procedures as specified in the protocol
- If female and not of childbearing potential, could be enrolled in the study. An FSH level may be measured at the discretion of the Investigator to confirm postmenopausal status
- If female and of childbearing potential, met all the following criteria to be enrolled in the study:
  - Had a negative pregnancy test at screening and on the day of vaccination (Day 1)
  - Had practiced adequate contraception or abstained from all activities that could result in pregnancy for at least 28 days prior to Day 1
  - Had agreed to continue adequate contraception through 3 months following vaccine administration. Adequate female contraception was defined as consistent and correct use of an FDA-approved contraceptive method in accordance with the product label
  - Was not currently breastfeeding

Participants who met any of the following criteria were excluded from Parts 2 or 3 of the study:

- Had close contact to someone with SARS-CoV-2 infection or COVID-19 as defined by the CDC in the 14 days prior to screening, unless the participant had been fully vaccinated for COVID-19 (Part 2), or in the 10 days prior to screening (Part 3)
- Was acutely ill or febrile (body temperature ≥38.0°C/100.4°F) 72 hours prior to or at screening or Day 1. Participants meeting this criterion could be rescheduled within the 28-day screening window and would retain their initially assigned participant number
- Had a medical, psychiatric, or occupational condition, or history of substance abuse that may pose additional risk as a result of participation or that could interfere with safety assessments or interpretation of results according to the Investigator’s judgment
- Had a current or previous diagnosis of immunocompromising/immunosuppressive condition, immune-mediated disease requiring immune-modifying therapy, asplenia, or recurrent severe infections (HIV-positive participants on antiretroviral therapy with cluster of differentiation 4 count ≥350 cells/mm^3^ and HIV-RNA ≤500 copies/mL within the past 12 months [Part 2] or 365 days [Part 3] were permitted)
- Had received systemic immunosuppressants or immune-modifying drugs for >14 days in total within 6 months (Part 2) or 180 days (Part 3) prior to screening (for corticosteroids ≥10 mg/day of prednisone or equivalent) or was anticipating the need for systemic immunosuppressive treatment at any time during participation in the study
- Had a history of anaphylaxis, urticaria, or other significant adverse reaction requiring medical intervention after receipt of a vaccine or any of the components contained in the investigational vaccine or the comparator vaccine, which is an egg-based influenza vaccine
- Had a coagulopathy or bleeding disorder considered a contraindication to intramuscular injection or phlebotomy
- Had received or planned to receive any licensed vaccine ≤28 days prior to vaccination (Day 1) or planned to receive a licensed vaccine within 28 days after vaccination, with the exception of vaccines authorized or approved for the prevention of COVID-19 (regardless of type of vaccine) that become available to participants during the study. Efforts were made to space study vaccination and COVID-19 vaccination by at least 7 and preferably 14 days, but COVID-19 vaccination should not have been delayed
- Had received a seasonal influenza vaccine or any other investigational influenza vaccine within 6 months prior to screening (Part 2) or within 180 days prior to randomization (Part 3)
- Had tested positive for influenza by CDC-recommended testing methods within 6 months (Part 2) or 180 days (Part 3) prior to screening
- Had received systemic immunoglobulins or blood products within 3 months (Part 2) or 90 days (Part 3) prior to screening or had plans to receive them during the study
- Had donated ≥450 mL of blood products within 28 days prior to screening or planned to donate blood products during the study
- Had participated in an interventional clinical study within 28 days prior to screening based on the medical history interview or planned to do so while participating in this study
- Was an immediate family member or household member of study personnel, study site staff, or Sponsor personnel

**Vaccine Strains**

In Part 1, mRNA-1010–encoded influenza strains included A/Wisconsin/588/2019(H1N1)pdm09, A/Hong Kong/45/2019(H3N2), B/Washington/02/2019 (B/Victoria lineage), and B/Phuket/3073/2013 (B/Yamagata lineage). In Parts 2 and 3, mRNA-1010–encoded influenza strains included A/Wisconsin/588/2019(H1N1)pdm09, A/Cambodia/e0826360/2020(H3N2), B/Washington/02/2019 (B/Victoria lineage), and B/Phuket/3073/2013 (B/Yamagata lineage). Afluria influenza strains included A/Victoria/2570/2019 IVR-215 (an A/Victoria/2570/2019 [H1N1]pdm09–like virus), A/Cambodia/e0826360/2020 IVR-224 (an A/Cambodia/e0826360/2020 [H3N2]–like virus), B/Victoria/705/2018 BVR-11 (a B/Washington/02/2019–like virus), and B/Phuket/3073/2013 BVR-1B (a B/Phuket/3073/2013–like virus).

**Hemagglutination Inhibition (HAI) Assay**

Sera from participants were tested by HAI assay using standard methods [1]. In brief, receptor destroying enzyme (RDE) was used to remove non-specific inhibitors of hemagglutination. Eleven 2-fold serial dilutions of treated serum samples were prepared in duplicate 96-well plates. Working dilutions of influenza virus equal to 4 HA units per well (per World Health Organization recommendations) were added to the serum dilutions and incubated for 60 minutes at room temperature. Guinea pig red blood cell (RBC) suspension (0.75%) was added to the serum-virus mixture and incubated for 60 minutes at room temperature. Following incubation, plates were read and the geometric mean titer (GMT) was calculated from duplicate plate readings. The HAI titer was defined as the last dilution at which agglutination of RBCs was inhibited [2]. Vaccine-heterologous H3N2 HAI responses were measured following a similar, standard HAI protocol.

***Ex vivo* Stimulation and Intracellular Staining Assay**

Peripheral blood mononuclear cell (PBMC) samples were prepared from whole blood specimens using standard ficoll separation methods and were cryopreserved and stored in liquid nitrogen vapor phase until testing. T-cell responses induced by vaccination in Part 1 were assessed by measuring cytokine production and expression of functional markers upon *ex vivo* stimulation with vaccine-specific peptide pools. Vaccine-matched lyophilized peptide pools (GeneScript Biotech Corp; Piscataway, NJ, USA) were composed of 15mers overlapping by 11 amino acids and were comprised of H1 from A/Wisconsin/588/2019(H1N1)pdm09, H3 from A/Hong Kong/45/2019(H3N2), B/Victoria from B/Washington/02/2019 (B/Victoria lineage), and B/Yamagata from B/Phuket/3073/2013 (B/Yamagata lineage). Following 6 hour *ex vivo* stimulation with protein transport inhibitor, PBMC samples were stained with fluorescently labeled antibodies for cell surface (CD4, CD8 CD14, CD16, CD19, CD56, CD45RA, CCR7, and TCRγδ) and intracellular (CD3, CD69, CD154, IL-2, IL-4, IL-5, IL-13, IL17a, IFN-γ, TNF-α, CD107a, and Granzyme B) markers of functional T-cell responses and acquired on a 5-laser Aurora spectral flow cytometer (Cytek^®^ Biosciences; Fremont, CA, USA). Data were analyzed using FCS-Express™ 7 IVD (De Novo Software; Pasadena, CA, USA).

**Sample Size**

Sample sizes for all parts of the study were not based on formal statistical hypothesis testing. In Part 1, it was expected that with 45 participants in each vaccine group, there was an approximately 90% probability to observe at least 1 participant with an adverse event (AE) if the true incidence rate of AEs was 5% or an approximately 99% probability if the true incidence rate was 10%. For Part 2, a sample size of 150 participants in each vaccine group had at least a 95% probability to observe at least 1 participant with an AE if the true incidence rate was 2%; if the true incidence rate was 3%, then the probability would be 99%. In Part 3, sample size of 50 participants in each vaccine group had at least a 92% probability to observe at least 1 participant with an AE if the true incidence rate was 5%; if the true incidence rate was 10%, then the probability would be 99%.

In Part 2 of the study, an analysis of covariance model was used with hemagglutination inhibition (HAI) titers at Day 29 as a dependent variable and a group variable as the fixed variable. The GMT of mRNA-1010 at Day 29 was estimated by the geometric least square mean (GLSM) from the model. The geometric mean ratio (GMTr; ratio of GMT) was estimated by the ratio of GLSM from the model. The corresponding 2-sided 95% confidence interval (CI) was calculated to assess the difference in immune response between mRNA-1010 compared with the active comparator at Day 29.

**Supplementary Results**

**Safety**

Three fatal events were reported during the study, and all were considered as unrelated to study vaccination by the study investigator. One participant aged 84 years in the placebo group died due to Stage IV renal cancer on Day 176. One participant aged 67 years in the mRNA-1010 100-µg group died due to cardiac arrest on Day 16; the participant had a history of diabetes mellitus, hypertension, and obesity. One participant aged 54 years in the mRNA-1010 6.25-µg group died due to a road traffic accident on Day 56.

**Supplemental Tables**

**Table S1.** Baseline Demographics for Participants in All Study Parts Combined (Safety Population)

|  | **18-49 years** | | | | | | | | **≥50 years** | | | | | | | |
| --- | --- | --- | --- | --- | --- | --- | --- | --- | --- | --- | --- | --- | --- | --- | --- | --- |
|  |  |  | **mRNA-1010** | | | | | |  |  | **mRNA-1010** | | | | | |
|  | **Placebo**  **(n = 21)** | **Afluria**  **(n = 56)** | **6.25 µg  (n = 35)** | **12.5 µg  (n = 34)** | **25 µg  (n = 94)** | **50 µg**  **(n = 81)** | **100 µg  (n = 80)** | **200 µg  (n = 23)** | **Placebo**  **(n = 45)** | **Afluria**  **(n = 104)** | **6.25 µg  (n = 50)** | **12.5 µg  (n = 50)** | **25 µg  (n = 200)** | **50 µg**  **(n = 192)** | **100 µg  (n = 192)** | **200 µg  (n = 45)** |
| **Age, y** |  |  |  |  |  |  |  |  |  |  |  |  |  |  |  |  |
| Mean ± SD | 38.1 ±8.27 | 34.3  ±9.39 | 32.0  ±8.78 | 33.3  ±8.31 | 33.2  ±8.88 | 34.7  ±9.00 | 33.6  ±9.40 | 37.3  ±8.27 | 64.2  ± 10.9 | 61.0  ±8.8 | 60.0  ±8.1 | 62.9  ±8.4 | 60.4  ±8.0 | 60.9  ±8.4 | 62.4  ±8.9 | 61.5  ±9.4 |
| Median (range) | 40.0  (18-49) | 37.0  (19-49) | 30.0  (18-49) | 33.0  (19-49) | 33.0  (18-49) | 36.0  (18-49) | 34.5 (18-49) | 39.0  (19-48) | 61.0  (50-90) | 58.0  (50-86) | 59.0  (50-75) | 59.0  (53-76) | 58.0  (50-86) | 59.0  (50-81) | 61.0  (50-83) | 59.0  (51-89) |
| **Sex, n (%)** |  |  |  |  |  |  |  |  |  |  |  |  |  |  |  |  |
| Male | 13 (61.9) | 30 (53.6) | 14 (40.0) | 13 (38.2) | 42 (44.7) | 38 (46.9) | 37 (46.3) | 6 (26.1) | 9 (37.5) | 21 (42.9) | 2 (13.3) | 10 (62.5) | 38 (36.2) | 55 (49.5) | 45 (39.8) | 5 (23.8) |
| Female | 8 (38.1) | 26 (46.4) | 21 (60.0) | 21 (61.8) | 52 (55.3) | 43 (53.1) | 43 (53.8) | 17 (73.9) | 15 (62.5) | 28 (57.1) | 13 (86.7) | 6 (37.5) | 67 (63.8) | 56 (50.5) | 68 (60.2) | 16 (76.2) |
| **Race, n (%)** |  |  |  |  |  |  |  |  |  |  |  |  |  |  |  |  |
| White | 16 (76.2) | 49 (87.5) | 32 (91.4) | 28 (82.4) | 75 (79.8) | 64 (79.0) | 65 (81.3) | 15 (65.2) | 22 (91.7) | 43 (87.8) | 12 (80.0) | 11 (68.8) | 90 (85.7) | 94 (84.7) | 100 (88.5) | 17 (81.0) |
| Black/African American | 4 (19.0) | 5 (8.9) | 2 (5.7) | 4 (11.8) | 10 (10.6) | 14 (17.3) | 9 (11.3) | 6 (26.1) | 1 (4.2) | 5 (10.2) | 2 (13.3) | 4 (25.0) | 11 (10.5) | 9 (8.1) | 11 (9.7) | 4 (19.0) |
| Asian | 1 (4.8) | 0 | 0 | 1 (2.9) | 2 (2.1) | 2 (2.5) | 2 (2.5) | 0 | 0 | 0 | 0 | 1 (6.3) | 0 | 2 (1.8) | 1 (0.9) | 0 |
| American Indian or Alaska Native | 0 | 0 | 0 | 0 | 2 (2.1) | 1 (1.2) | 0 | 0 | 0 | 0 | 1 (6.7) | 0 | 0 | 1 (0.9) | 0 | 0 |
| Native Hawaiian or Other Pacific Islander | 0 | 0 | 0 | 0 | 0 | 0 | 0 | 0 | 0 | 0 | 0 | 0 | 1 (1.0) | 0 | 0 | 0 |
| Multiracial | 0 | 2 (3.6) | 1 (2.9) | 1 (2.9) | 3 (3.2) | 0 | 1 (1.3) | 0 | 0 | 0 | 0 | 0 | 1 (1.0) | 3 (2.7) | 0 | 0 |
| Other | 0 | 0 | 0 | 0 | 1 (1.1) | 0 | 1 (1.3) | 0 | 0 | 0 | 0 | 0 | 1 (1.0) | 1 (0.9) | 0 | 0 |
| Not Reported | 0 | 0 | 0 | 0 | 1 (1.1) | 0 | 2 (2.5) | 2 (8.7) | 1 (4.2) | 1 (2.0) | 0 | 0 | 1 (1.0) | 1 (0.9) | 1 (0.9) | 0 |
| Unknown | 0 | 0 | 0 | 0 | 0 | 0 | 0 | 0 | 0 | 0 | 0 | 0 | 0 | 0 | 0 | 0 |
| **Ethnicity, n (%)** |  |  |  |  |  |  |  |  |  |  |  |  |  |  |  |  |
| Hispanic or Latino | 4 (19.0) | 17 (30.4) | 8 (22.9) | 11 (32.4) | 22 (23.4) | 27 (33.3) | 22 (27.5) | 4 (17.4) | 1 (4.2) | 12 (24.5) | 4 (26.7) | 3 (18.8) | 16 (15.2) | 17 (15.3) | 16 (14.2) | 4 (19.0) |
| Not Hispanic or Latino | 17 (81.0) | 39 (69.9) | 27 (77.1) | 22 (64.7) | 70 (74.5) | 54 (66.7) | 56 (70.0) | 18 (78.3) | 22 (91.7) | 37 (75.5) | 9 (60.0) | 13 (81.3) | 88 (83.8) | 93 (83.8) | 95 (84.1) | 17 (81.0) |
| Not Reported | 0 | 0 | 0 | 1 (2.9) | 1 (1.1) | 0 | 1 (1.3) | 1 (4.3) | 0 | 0 | 2 (13.3) | 0 | 1 (1.0) | 1 (0.9) | 2 (1.8) | 0 |
| Unknown | 0 | 0 | 0 | 0 | 1 (1.1) | 0 | 1 (0.3) | 0 | 1 (4.2) | 0 | 0 | 0 | 0 | 0 | 0 | 0 |
| **Weight, kg** |  |  |  |  |  |  |  |  |  |  |  |  |  |  |  |  |
| Mean ± SD | 84.8 ±16.3 | 88.6 ±25.6 | 94.4 ±31.0 | 90.0 ±17.0 | 88.4 ±23.4 | 89.1 ±22.9 | 89.2 ±24.0 | 76.5 ±14.8 | 78.6 ±14.6 | 85.5 ±19.9 | 86.6 ±23.6 | 85.4 ±17.6 | 87.9 ±19.4 | 88.1 ±20.6 | 86.5 ±23.6 | 75.8 ±10.0 |
| **Height, cm** |  |  |  |  |  |  |  |  |  |  |  |  |  |  |  |  |
| Mean ± SD | 172.0 ±9.4 | 170.5 ±13.6 | 171.8 ±11.4 | 169.6 ±11.0 | 170.2 ±10.5 | 169.9 ±8.7 | 170.5 ±10.0 | 166.6 ±8.7 | 168.9 ±8.4 | 167.0 ±9.6 | 165.9 ±7.2 | 171.6 ±8.6 | 168.4 ±8.5 | 169.6 ±10.4 | 168.9 ±9.7 | 166.0 ±7.9 |
| **BMI, kg/m^2^** |  |  |  |  |  |  |  |  |  |  |  |  |  |  |  |  |
| Mean ± SD | 28.6 ±4.6 | 30.3 ±7.3 | 31.8 ±9.5 | 31.3 ±5.4 | 30.6 ±8.1 | 30.9 ±7.7 | 30.7 ±7.9 | 27.5 ±4.3 | 27.4 ±4.1 | 30.7 ±6.6 | 31.5 ±8.1 | 29.2 ±7.3 | 30.9 ±6.1 | 30.6 ±6.3 | 30.2 ±7.3 | 27.6 ±3.9 |
| **Received seasonal influenza vaccine in prior season, n (%)^a^** |  |  |  |  |  |  |  |  |  |  |  |  |  |  |  |  |
| Yes | 7 (33.3) | 11 (19.6) | 2 (5.7) | 0 | 17 (18.1) | 20 (24.7) | 25 (31.3) | 6 (26.1) | 12 (50.0) | 16 (32.7) | 2 (13.3) | 3 (18.8) | 39 (37.1) | 45 (40.5) | 53 (46.9) | 14 (66.7) |
| No | 13 (61.9) | 45 (80.4) | 33 (94.3) | 34 (100) | 77 (81.9) | 61 (75.3) | 54 (67.5) | 17 (73.9) | 9 (37.5) | 33 (67.3) | 13 (86.7) | 13 (81.3) | 66 (62.9) | 64 (57.7) | 57 (50.4) | 6 (28.6) |
| Unknown | 1 (4.8) | 0 | 0 | 0 | 0 | 0 | 1 (1.3) | 0 | 3 (12.5) | 0 | 0 | 0 | 0 | 2 (1.8) | 3 (2.7) | 1 (4.8) |

The safety population included all randomized participants who received vaccination.

BMI, body mass index; mRNA, messenger RNA; SD, standard deviation.

^a^Participants were excluded from the study if a seasonal influenza vaccine or any other investigational influenza vaccine was received after January 1,2021 (Part 1), within 6 months prior to screening (Part 2), or within 180 days prior to randomization (Part 3). For Part 1, a participant was considered to have received seasonal influenza vaccine in the prior season if received between August 2020 to December 2020; for Part 2, a participant was considered to have received seasonal influenza vaccine if received since September 2020; for Part 3, a participant was considered to have received seasonal influenza vaccine if received since September 2021.

**Table S2.** Summary of Unsolicited Treatment-Emergent Adverse Events for All Study Parts Combined (Safety Population)

|  | **18-49 years** | | | | | | | | **≥50 years** | | | | | | | |
| --- | --- | --- | --- | --- | --- | --- | --- | --- | --- | --- | --- | --- | --- | --- | --- | --- |
|  |  | | **mRNA-1010** | | | | | |  | | **mRNA-1010** | | | | | |
|  | **Placebo**  **(n = 21)** | **Afluria**  **(n = 56)** | **6.25 µg  (n = 35)** | **12.5 µg  (n = 34)** | **25 µg  (n = 94)** | **50 µg**  **(n = 81)** | **100 µg  (n = 80)** | **200 µg  (n = 23)** | **Placebo**  **(n = 45)** | **Afluria**  **(n = 104)** | **6.25 µg  (n = 50)** | **12.5 µg  (n = 50)** | **25 µg  (n = 200)** | **50 µg**  **(n = 192)** | **100 µg  (n = 192)** | **200 µg  (n = 45)** |
| **Within 28 days of vaccination** |  |  |  |  |  |  |  |  |  |  |  |  |  |  |  |  |
| **All unsolicited TEAEs, n (%)** |  |  |  |  |  |  |  |  |  |  |  |  |  |  |  |  |
| All | 2 (9.5) | 11 (19.6) | 13 (37.1) | 11 (32.4) | 19 (20.2) | 16 (19.8) | 18 (22.5) | 6 (26.1) | 4 (16.7) | 9 (18.4) | 4 (26.7) | 3 (18.8) | 28 (26.7) | 26 (23.4) | 28 (24.8) | 4 (19.0) |
| SAEs | 0 | 0 | 0 | 0 | 0 | 0 | 1 (1.3) | 0 | 1 (4.2) | 0 | 1 (6.7) | 0 | 0 | 1 (0.9) | 1 (0.9) | 0 |
| Fatal | 0 | 0 | 0 | 0 | 0 | 0 | 0 | 0 | 0 | 0 | 0 | 0 | 0 | 0 | 1 (0.9) | 0 |
| MAAEs | 0 | 3 (5.4) | 5 (14.3) | 2 (5.9) | 9 (9.6) | 2 (2.5) | 8 (10.0) | 2 (8.7) | 3 (12.5) | 3 (6.1) | 3 (20.0) | 3 (18.8) | 9 (8.6) | 8 (7.2) | 11 (9.7) | 2 (9.5) |
| Leading to dose delay | 0 | 0 | 0 | 0 | 0 | 0 | 0 | 0 | 0 | 0 | 0 | 0 | 0 | 0 | 0 | 0 |
| Leading to vaccination withdrawal | 0 | 0 | 0 | 0 | 0 | 0 | 0 | 0 | 0 | 0 | 0 | 0 | 0 | 0 | 0 | 0 |
| Leading to study discontinuation | 0 | 0 | 0 | 0 | 0 | 0 | 0 | 0 | 0 | 0 | 0 | 0 | 0 | 0 | 1 (0.9) | 0 |
| Severe | 0 | 0 | 1 (2.9) | 1 (2.9) | 2 (2.1) | 2 (2.5) | 3 (3.8) | 0 | 0 | 0 | 0 | 0 | 1 (1.0) | 0 | 2 (1.8) | 0 |
| AESIs | 0 | 0 | 0 | 0 | 0 | 0 | 0 | 0 | 0 | 0 | 0 | 0 | 0 | 0 | 0 | 0 |
| **Related unsolicited TEAEs, n (%)** |  |  |  |  |  |  |  |  |  |  |  |  |  |  |  |  |
| All | 2 (9.5) | 4 (7.1) | 6 (17.1) | 7 (20.6) | 8 (8.5) | 5 (6.2) | 5 (6.3) | 2 (8.7) | 0 | 3 (6.1) | 0 | 0 | 11 (10.5) | 10 (9.0) | 10 (8.8) | 1 (4.8) |
| SAEs | 0 | 0 | 0 | 0 | 0 | 0 | 0 | 0 | 0 | 0 | 0 | 0 | 0 | 0 | 0 | 0 |
| Fatal | 0 | 0 | 0 | 0 | 0 | 0 | 0 | 0 | 0 | 0 | 0 | 0 | 0 | 0 | 0 | 0 |
| MAAEs | 0 | 0 | 0 | 1 (2.9) | 2 (2.1) | 0 | 0 | 0 | 0 | 0 | 0 | 0 | 0 | 0 | 1 (0.9) | 0 |
| Leading to dose delay | 0 | 0 | 0 | 0 | 0 | 0 | 0 | 0 | 0 | 0 | 0 | 0 | 0 | 0 | 0 | 0 |
| Leading to vaccination withdrawal | 0 | 0 | 0 | 0 | 0 | 0 | 0 | 0 | 0 | 0 | 0 | 0 | 0 | 0 | 0 | 0 |
| Leading to study discontinuation | 0 | 0 | 0 | 0 | 0 | 0 | 0 | 0 | 0 | 0 | 0 | 0 | 0 | 0 | 0 | 0 |
| Severe | 0 | 0 | 1 (2.9) | 1 (2.9) | 1 (1.1) | 2 (2.5) | 2 (2.5) | 0 | 0 | 0 | 0 | 0 | 1 (1.0) | 0 | 1 (0.9) | 0 |
| AESIs | 0 | 0 | 0 | 0 | 0 | 0 | 0 | 0 | 0 | 0 | 0 | 0 | 0 | 0 | 0 | 0 |
| **Within 6 months of vaccination** |  |  |  |  |  |  |  |  |  |  |  |  |  |  |  |  |
| **All unsolicited TEAEs, n (%)** |  |  |  |  |  |  |  |  |  |  |  |  |  |  |  |  |
| SAEs | 0 | 2 (3.6) | 0 | 0 | 1 (1.1) | 1 (1.2) | 1 (1.3) | 1 (4.3) | 2 (8.3) | 0 | 2 (13.3) | 0 | 2 (1.9) | 4 (3.6) | 3 (2.7) | 0 |
| Fatal | 0 | 0 | 0 | 0 | 0 | 0 | 0 | 0 | 1 (4.2) | 0 | 1 (6.7) | 0 | 0 | 0 | 1 (0.9) | 0 |
| MAAEs | 2 (9.5) | 10 (17.9) | 11 (31.4) | 9 (26.5) | 22 (23.4) | 12 (14.8) | 26 (32.5) | 6 (26.1) | 8 (33.3) | 17 (34.7) | 7 (46.7) | 6 (37.5) | 36 (34.3) | 38 (34.2) | 36 (31.9) | 6 (28.6) |
| Leading to study discontinuation | 0 | 0 | 0 | 0 | 0 | 0 | 0 | 0 | 1 (4.2) | 0 | 1 (6.7) | 0 | 0 | 0 | 1 (0.9) | 0 |
| **Related unsolicited TEAEs, n (%)** |  |  |  |  |  |  |  |  |  |  |  |  |  |  |  |  |
| SAEs | 0 | 0 | 0 | 0 | 0 | 0 | 0 | 0 | 0 | 0 | 0 | 0 | 0 | 0 | 0 | 0 |
| Fatal | 0 | 0 | 0 | 0 | 0 | 0 | 0 | 0 | 0 | 0 | 0 | 0 | 0 | 0 | 0 | 0 |
| MAAEs | 0 | 0 | 0 | 1 (2.9) | 2 (2.1) | 0 | 0 | 0 | 0 | 0 | 0 | 0 | 0 | 1 (0.9) | 0 | 1 (0.4) |
| Leading to study discontinuation | 0 | 0 | 0 | 0 | 0 | 0 | 0 | 0 | 0 | 0 | 0 | 0 | 0 | 0 | 0 | 0 |

A TEAE was defined as any event not present before study vaccination or any event already present that worsened in intensity or frequency for up to 6 months after vaccination.

AE, adverse event; AESI, adverse event of special interest; MAAE, medically attended adverse event; SAE, serious adverse event; TEAE, treatment-emergent adverse event.

**Table S3.** Summary of Influenza Antibody Responses by HAI Through Day 181 by Age Group in Study Part 1 (Per-Protocol Population)

|  | **Part 1** | | | | | | | | |
| --- | --- | --- | --- | --- | --- | --- | --- | --- | --- |
|  | **18-49 Years** | | | | | **≥50 Years** | | | |
|  | **Placebo**  **(n=19)** | **50 µg mRNA-1010**  **(n=22)** | **100 µg mRNA-1010 (n=21)** | | **200 µg mRNA-1010 (n=22)** | **Placebo**  **(n=24)** | **50 µg mRNA-1010**  **(n=22)** | **100 µg mRNA-1010 (n=25)** | **200 µg mRNA-1010 (n=21)** |
| **Influenza A/H1N1 Antibodies by HAI Assay** | | | | | | | | | |
| **Baseline  (Day 1)** |  |  |  | |  |  |  |  |  |
| n^a^ | 19 | 22 | 21 | | 22 | 22 | 21 | 23 | 19 |
| GMT (95% CI) | 65.4 (34.5- 123.8) | 55.6 (30.3-102.3) | 51.2 (32.6-80.2) | | 59.2 (36.4-96.4) | 46.0 (25.8-81.9) | 53.8 (33.6-86.2) | 29.1 (19.8-43.0) | 48.8 (26.1-91.0) |
| **Day 8** |  |  |  | |  |  |  |  |  |
| n^b^ | 19 | 21 | 21 | | 22 | 22 | 21 | 23 | 19 |
| GMT (95% CI) | 48.9 (24.5-97.4) | 280.5 (151.2-520.4) | 320.1 (160.4-638.9) | | 345.9 (179.8-665.6) | 36.9 (21.7-62.7) | 122.8 (76.9-196.1) | 88.9 (49.1-160.9) | 292.1 (161.3-529.2) |
| GMFR (95% CI) | 0.8 (0.6-1.0) | 5.3 (2.9-9.8) | 6.3 (3.3-11.8) | | 5.8 (3.2-10.8) | 0.8 (0.6-1.1) | 2.3 (1.4-3.6) | 3.0 (1.6-5.7) | 6.0 (3.1-11.7) |
| Seroconversion,^c^ n^d^ (%) [95% CI] | 1 (5.3) [0.1-26.0] | 14 (66.7) [43.0-85.4] | 15 (71.4) [47.8-88.7] | | 14 (63.6) [40.7-82.8] | 1 (4.5) [0.1-22.8] | 7 (33.3) [14.6-57.0] | 10 (43.5) [23.2-65.5] | 13 (68.4) [43.4-87.4] |
| **Day 29** |  |  |  | |  |  |  |  |  |
| n^b^ | 19 | 22 | 21 | | 22 | 22 | 21 | 23 | 19 |
| GMT (95% CI) | 66.6 (34.5-128.9) | 537.9 (265.4-1090.4) | 572.9 (288.0-1139.7) | | 785.7 (419.9-1470.0) | 52.3 (30.7-89.0) | 309.6 (173.8-551.4) | 200.6 (120.9-333.0) | 436.3 (200.1-951.3) |
| GMFR (95% CI) | 1.0 (0.8-1.2) | 9.7 (5.3-17.7) | 11.2 (6.2-20.2) | | 13.3 (8.6-20.6) | 1.1 (0.8-1.5) | 5.8 (3.3-10.1) | 6.9 (4.3-11.0) | 9.0 (4.6-17.5) |
| Seroconversion,^c^ n^d^ (%) [95% CI] | 0 [0-17.6] | 17 (77.3) [54.6-92.2] | 19 (90.5) [69.6-98.8] | | 21 (95.5) [77.2-99.9] | 2 (9.1) [1.1-29.2] | 12 (57.1) [34.0-78.2] | 17 (73.9) [51.6-89.8] | 16 (84.2) [60.4-96.6] |
| **Day 181** |  |  |  | |  |  |  |  |  |
| n^b^ | 18 | 20 | 18 | | 20 | 21 | 19 | 23 | 16 |
| GMT (95% CI) | 57.6 (26.8-123.9) | 207.5 (126.7-339.8) | 111.0 (59.4-207.1) | | 165.5 (85.4-320.9) | 77.4 (40.1-149.2) | 133.4 (86.8-204.8) | 54.1 (36.5-80.0) | 153.2 (85.8-273.5) |
| GMFR (95% CI) | 0.8 (0.6-1.2) | 3.5 (1.8-7.0) | 2.7 (1.4-5.2) | | 3.1 (2.0-4.7) | 1.6 (1.0-2.7) | 2.5 (1.4-4.4) | 1.9 (1.2-2.9) | 2.7 (1.6-4.7) |
| Seroconversion,^c^ n^d^ (%) [95% CI] | 0 [0-18.5] | 9 (45.0) [23.1-68.5] | 7 (38.9) [17.3-64.3] | | 6 (30.0) [11.9-54.3] | 5 (23.8) [8.2-47.2] | 8 (42.1) [20.2-66.5] | 7 (30.4) [13.2-52.9] | 6 (37.5) [15.2-64.6] |
| **Influenza A/H3N2 Antibodies by HAI Assay** | | | | | | | | | |
| **Baseline  (Day 1)** |  |  |  | |  |  |  |  |  |
| n^a^ | 19 | 22 | 21 | | 22 | 22 | 21 | 23 | 19 |
| GMT (95% CI) | 69.0 (40.4-118.0) | 66.1 (40.3-108.2) | 59.4 (41.9-84.0) | | 90.7 (51.6-159.1) | 43.2 (24.9-74.9) | 41.3 (25.3-67.4) | 43.7 (27.3-70.0) | 60.8 (32.1-115.0) |
| **Day 8** |  |  |  | |  |  |  |  |  |
| n^b^ | 19 | 21 | 21 | | 22 | 22 | 21 | 23 | 19 |
| GMT (95% CI) | 74.3 (46.5-118.7) | 294.5 (169.7-511.1) | 309.7 (190.0-504.8) | | 315.0 (209.2-474.3) | 37.5 (24.4-57.4) | 152.3 (97.3-238.2) | 162.4 (97.4-270.6) | 384.0 (259.7-567.9) |
| GMFR (95% CI) | 1.1 (0.7-1.6) | 4.8 (2.5-9.2) | 5.2 (3.0-9.2) | | 3.5 (1.8-6.6) | 0.9 (0.6-1.3) | 3.7 (2.1-6.6) | 3.7 (1.7-8.0) | 6.3 (3.2-12.6) |
| Seroconversion,^c^ n^d^ (%) [95% CI] | 1 (5.3) [0.1-26.0] | 11 (52.4) [29.8-74.3] | 11 (52.4) [29.8-74.3] | | 13 (59.1) [36.4-79.3] | 0 [0-15.4] | 9 (42.9) [21.8-66.0] | 13 (56.5) [34.5-76.8] | 13 (68.4) [43.4-87.4] |
| **Day 29** |  |  |  | |  |  |  |  |  |
| n^b^ | 19 | 22 | 21 | | 22 | 22 | 21 | 23 | 19 |
| GMT (95% CI) | 81.3 (45.1-146.6) | 529.7 (350.5-800.4) | 371.0 (208.3-660.9) | | 674.2 (424.8-1070.0) | 41.2 (24.4-69.6) | 262.6 (162.1-425.4) | 288.1 (197.8-419.7) | 413.1 (253.5-673.0) |
| GMFR (95% CI) | 1.2 (0.6-2.2) | 8.0 (5.1-12.7) | 6.2 (3.5-11.3) | | 7.4 (4.9-11.4) | 1.0 (0.8-1.1) | 6.4 (3.9-10.4) | 6.6 (4.3-10.2) | 6.8 (3.9-12.0) |
| Seroconversion,^c^ n^d^ (%) [95% CI] | 1 (5.3) [0.1-26.0] | 18 (81.8) [59.7-94.8] | 16 (76.2) [52.8-91.8] | | 17 (77.3) [54.6-92.2] | 0 [0-15.4] | 17 (81.0) [15.1-94.6] | 17 (73.9) [51.6-89.8] | 14 (73.7) [48.8-90.8] |
| **Day 181** |  |  |  | |  |  |  |  |  |
| n^b^ | 18 | 20 | 18 | | 20 | 21 | 19 | 23 | 16 |
| GMT (95% CI) | 88.0 (44.7-173.2) | 222.3 (130.2-379.9) | 190.5 (110.8-327.5) | | 246.6 (154.9-392.6) | 53.8 (32.3-89.6) | 154.2 (109.0-218.3) | 137.6 (93.8-201.8) | 221.4 (126.2-388.5) |
| GMFR (95% CI) | 1.3 (0.7-2.4) | 3.3 (2.1-5.1) | 3.4 (1.9-6.4) | | 2.8 (1.7-4.6) | 1.3 (0.8-2.0) | 3.2 (2.0-5.1) | 3.2 (2.0-5.1) | 3.5 (1.9-6.4) |
| Seroconversion,^c^ n^d^ (%) [95% CI] | 2 (11.1) [1.4-34.7] | 9 (45.0) [23.1-68.5] | 6 (33.3) [13.3-59.0] | | 10 (50.0) [27.2-72.8] | 3 (14.3) [3.0-36.3] | 9 (47.4) [24.4-71.1] | 12 (52.2) [30.6-73.2] | 8 (50.0) [24.6-75.4] |
| **Influenza B/Victoria Lineage Antibodies by HAI Assay** | | | | | | | | | |
| **Baseline  (Day 1)** |  |  |  | |  |  |  |  |  |
| n^a^ | 19 | 22 | 21 | | 22 | 22 | 21 | 23 | 18 |
| GMT (95% CI) | 143.3 (82.5-248.8) | 113.1 (65.7-194.6) | 109.4 (66.6-179.8) | | 136.7 (89.7-208.2) | 93.5 (51.7-169.1) | 131.3 (83.4-206.6) | 77.6 (46.5-129.4) | 139.6 (70.5-276.4) |
| **Day 8** |  |  |  | |  |  |  |  |  |
| n^b^ | 19 | 21 | 21 | | 22 | 22 | 21 | 23 | 19 |
| GMT (95% CI) | 46.3 (28.9-74.1) | 92.8 (58.8-146.5) | 122.9 (82.2-183.7) | | 102.9 (65.8-161.1) | 27.0 (15.2-47.8) | 89.8 (60.5-133.2) | 104.9 (61.8-178.0) | 148.8 (91.4-242.2) |
| GMFR (95% CI) | 0.3 (0.2-0.5) | 0.9 (0.5-1.4) | 1.1 (0.6-2.0) | | 0.8 (0.4-1.4) | 0.3 (0.2-0.4) | 0.7 (0.4-1.2) | 1.4 (0.8-2.2) | 1.0 (0.5-2.0) |
| Seroconversion,^c^ n^d^ (%) [95% CI] | 0 [0-17.6] | 2 (9.5) [1.2-30.4] | 4 (19.0) [5.4-41.9] | | 2 (9.1) [1.1-29.2] | 0 [0-15.4] | 1 (4.8) [0.1-23.8] | 4 (17.4) [5.0-38.8] | 3 (16.7) [3.6-41.2] |
| **Day 29** |  |  |  | |  |  |  |  |  |
| n^b^ | 19 | 22 | 21 | | 22 | 22 | 21 | 23 | 19 |
| GMT (95% CI) | 126.1 (75.7-210.2) | 260.7 (168.5-403.3) | 179.6 (117.5-274.5) | | 313.2 (217.9-450.1) | 99.6 (54.2-183.2) | 215.3 (145.2-319.2) | 161.6 (94.8-275.4) | 247.8 (157.7-389.4) |
| GMFR (95% CI) | 0.88 (0.6-1.4) | 2.3 (1.7-3.1) | 1.6 (1.0-2.7) | | 2.3 (1.6-3.2) | 1.1 (0.9-1.2) | 1.6 (1.3-2.1) | 2.1 (1.4-3.2) | 1.8 (1.0-3.1) |
| Seroconversion,^c^ n^d^ (%) [95% CI] | 1 (5.3) [0.1-26.0] | 4 (18.2) [5.2-40.3] | 4 (19.0) [5.4-41.9] | | 6 (27.3) [10.7-50.2] | 0 [0-15.4] | 2 (9.5) [1.2-30.4] | 3 (13.0) [2.8-33.6] | 4 (22.2) [6.4-47.6] |
| **Day 181** |  |  |  | |  |  |  |  |  |
| n^b^ | 18 | 20 | 18 | | 20 | 21 | 19 | 23 | 16 |
| GMT (95% CI) | 42.4 (26.6-67.6) | 74.7 (46.8-119.1) | 57.7 (38.7-86.0) | | 69.7 (39.8-122.1) | 33.9 (18.9-60.8) | 78.6 (47.9-129.1) | 86.2 (55.5-133.9) | 71.8 (40.9-126.0) |
| GMFR (95% CI) | 0.3 (0.2-0.5) | 0.6 (0.3-1.0) | 0.6 (0.3-0.9) | | 0.5 (0.3-0.8) | 0.4 (0.2-0.6) | 0.5 (0.3-0.9) | 1.1 (0.7-1.7) | 0.4 (0.2-0.8) |
| Seroconversion,^c^ n^d^ (%) [95% CI] | 0 [0-18.5] | 2 (10.0) [1.2-31.7] | 1 (5.6) [0.1-27.3] | | 1 (5.0) [0.1-24.9] | 0 [0-16.1] | 1 (5.3) [0.1-26.0] | 5 (21.7) [7.5-43.7] | 1 (6.7) [0.2-32.0] |
| **Influenza B/Yamagata Lineage Antibodies by HAI Assay** | | | | | | | | | |
| **Baseline (Day 1)** |  |  |  |  | |  |  |  |  |
| n^a^ | 19 | 22 | 21 | | 22 | 22 | 21 | 23 | 19 |
| GMT (95% CI) | 157.1 (79.7-309.6) | 159.9 (91.9-278.1) | 137.8 (75.1-252.7) | | 181.6 (99.4-331.7) | 85.2 (42.2-171.8) | 96.0 (58.2-158.2) | 54.0 (31.4-92.8) | 86.0 (42.4-174.6) |
| **Day 8** |  |  |  | |  |  |  |  |  |
| n^b^ | 19 | 21 | 21 | | 22 | 22 | 21 | 23 | 19 |
| GMT (95% CI) | 78.6 (39.6-156.0) | 152.2 (98.6-234.7) | 182.6 (108.3-307.8) | | 260.8 (172.2-394.9) | 40.0 (24.1-66.2) | 92.8 (67.8-127.0) | 77.8 (48.6-124.2) | 138.3 (85.0-224.9) |
| GMFR (95% CI) | 0.5 (0.3-0.7) | 1.0 (0.7-1.4) | 1.3 (0.8-2.2) | | 1.4 (0.8-2.4) | 0.5 (0.3-0.7) | 1.0 (0.6-1.6) | 1.4 (0.9-2.3) | 1.6 (0.9-2.8) |
| Seroconversion,^c^ n^d^ (%) [95% CI] | 0 [0-17.6] | 2 (9.5) [1.2-30.4] | 4 (19.0) [5.4-41.9] | | 3 (13.6) [2.9-34.9] | 0 [0-15.4] | 2 (9.5) [1.2-30.4] | 3 (13.0) [2.8-33.6] | 3 (15.8) [3.4-39.6] |
| **Day 29** |  |  |  | |  |  |  |  |  |
| n^b^ | 19 | 22 | 21 | | 22 | 22 | 21 | 23 | 19 |
| GMT (95% CI) | 143.3 (77.0-266.5) | 467.1 (295.0-739.5) | 383.7 (201.9-729.2) | | 610.5 (417.4-892.9) | 78.8 (41.2-150.5) | 304.5 (195.3-474.9) | 269.6 (169.4-429.0) | 325.8 (211.3-502.4) |
| GMFR (95% CI) | 0.9 (0.7-1.2) | 2.9 (2.0-4.2) | 2.8 (1.8-4.2) | | 3.4 (1.9-5.9) | 0.9 (0.8-1.0) | 3.2 (2.1-4.8) | 5.0 (3.0-8.4) | 3.8 (2.3-6.2) |
| Seroconversion,^c^ n^d^ (%) [95% CI] | 1 (5.3) [0.1-26.0] | 9 (40.9) [20.7- 63.6] | 9 (42.9) [21.8-66.0]) | | 12 (54.5) [32.2-75.6] | 0 [0-15.4] | 9 (42.9) [21.8-66.0] | 13 (56.0) [34.5-76.8) | 9 (47.4) [24.4-71.1) |
| **Day 181** |  |  |  | |  |  |  |  |  |
| n^b^ | 18 | 20 | 18 | | 20 | 21 | 19 | 23 | 16 |
| GMT (95% CI) | 63.6 (31.9-126.7) | 121.2 (76.0) | 83.1 (48.7-142.0) | | 144.2 (86.1-241.6) | 48.7 (28.0-84.7) | 92.5 (65.2-131.2) | 61.0 (44.9-82.9) | 81.7 (39.6-168.7) |
| GMFR (95% CI) | 0.4 (0.3-0.6) | 0.7 (0.4-1.1) | 0.8 (0.5-1.3) | | 0.9 (0.6-1.2) | 0.6 (0.4-1.0) | 0.9 (0.5-1.5) | 1.1 (0.7-1.9) | 0.8 (0.6-1.3) |
| Seroconversion,^c^ n^d^ (%) [95% CI] | 0 [0-18.5] | 2 (10.0) [1.2-31.7] | 2 (11.1) [1.4-34.7] | | 1 (5.0) [0.1-24.9] | 0 [0-16.1] | 1 (5.3) [0.1-26.0] | 3 (13.0) [2.8-33.6] | 0 [0-20.6] |

The per-protocol population comprised all randomly assigned participants who received the vaccination and who did not have influenza infection at baseline through Day 29 and had no major protocol deviations that could impact the immune response.

CI, confidence interval; GMFR, geometric mean fold rise; GMT, geometric mean titer; HA, hemagglutinin; HAI, hemagglutination inhibition; mRNA, messenger RNA.

^a^Number of subjects with non-missing baseline (Day 1) anti-HA antibody data.

^b^Number of subjects with non-missing anti-HA antibody data at the corresponding visit.

^c^Seroconversion at a subject level is defined as a post-baseline titer ≥1:40 if baseline is <1:10 or a 4-fold or greater rise if baseline is ≥1:10 in anti-HA antibodies.

^d^Number of subjects meeting the criterion at the corresponding visit. Percentage is based on the number of subjects with non-missing baseline (Day 1) anti-HA antibody (n^b^ rows).

**Table S4.** Summary of Influenza Antibody Responses by HAI Through Day 181 by Age Group in Study Part 2 (Per-Protocol Population)

|  | **Part 2** | | | | | | | | | | | |
| --- | --- | --- | --- | --- | --- | --- | --- | --- | --- | --- | --- | --- |
|  | **18-49 Years** | | | | **50-64 Years** | | | | **≥65 Years** | | | |
|  | **Afluria**  **(n=20)** | **25 µg  mRNA-1010**  **(n=55)** | **50 µg  mRNA-1010 (n=53)** | **100 µg  mRNA-1010 (n=57)** | **Afluria**  **(n=20)** | **25 µg mRNA-1010**  **(n=58)** | **50 µg mRNA-1010 (n=54)** | **100 µg mRNA-1010 (n=54)** | **Afluria**  **(n=12)** | **25 µg  mRNA-1010**  **(n=31)** | **50 µg mRNA-1010 (n=31)** | **100 µg mRNA-1010 (n=30)** |
| **Influenza A/H1N1 Antibodies by HAI Assay** | | | | | | | | | | | | |
| **Baseline  (Day 1)** |  |  |  |  |  |  |  |  |  |  |  |  |
| n^a^ | 20 | 55 | 53 | 57 | 20 | 58 | 54 | 54 | 12 | 31 | 31 | 30 |
| GMT (95% CI) | 45.1 (25.0, 81.4) | 55.5 (42.3, 72.8) | 39.2 (27.6, 55.6) | 56.9 (42.6, 76.1) | 47.6 (23.9, 95.3) | 36.3 (26.9, 50.0) | 28.7 (20.8, 39.4) | 34.9 (26.2, 46.6) | 38.8 (17.3, 87.3) | 48.9 (34.0, 70.2) | 54.7 (34.9, 85.7) | 29.2 (21.5, 39.8) |
| **Day 29** |  |  |  |  |  |  |  |  |  |  |  |  |
| n^b^ | 20 | 55 | 53 | 57 | 20 | 58 | 54 | 54 | 12 | 31 | 31 | 30 |
| GMT (95% CI) | 246.7 (12.2, 471.0) | 591.8 (428.5, 817.3) | 613.7 (434.9, 866.0) | 805.6 (588.4, 1103.0) | 171.3 (102.9, 285.3) | 262.8 (191.2, 361.2) | 418.8 (295.7, 593.1) | 418.0 (301.7, 579.1) | 134.5 (59.2, 305.9) | 228.1 (133.2, 390.5) | 311.7 (187.5, 518.4) | 253.9 (157.7, 408.8) |
| GMFR (95% CI) | 5.5 (2.3, 13.0) | 10.7 (7.9, 14.5) | 15.7 (9.8, 24.9) | 14.2 (10.2, 19.6) | 3.6 (2.3, 5.7) | 7.2 (5.2, 10.1) | 14.6 (10.1, 12.1) | 12.0 (8.7, 16.4) | 3.5 (1.6, 7.7) | 4.7 (2.9, 7.5) | 5.7 (3.5, 9.4) | 8.7 (5.6, 13.4) |
| Seroconversion,^c^ n^d^ (%) [95% CI] | 17 (85.0) [62.1, 96.8] | 48 (87.3) [75.5, 94.7] | 48 (90.6) [79.3, 96.9] | 49 (86.0) [74.2, 93.7] | 9 (45.0) [23.1, 68.5] | 40 (69.0) [55.5, 80.5] | 47 (87.0) [75.1, 94.6] | 49 (90.7) [79.7, 96.9] | 5 (41.7) [15.2, 72.3] | 16 (51.6) [33.1, 69.9] | 22 (71.0) [52.0, 85.8] | 23 (76.7) [57.7, 90.1] |
| **Day 91** |  |  |  |  |  |  |  |  |  |  |  |  |
| n^b^ | 18 | 51 | 52 | 55 | 20 | 56 | 51 | 54 | 12 | 30 | 31 | 29 |
| GMT (95% CI) | 157.0 (75.7, 325.8) | 259.3 (182.7, 367.8) | 301.3 (214.8, 422.7) | 360.8 (271.7, 479.0) | 111.2 (55.9, 221.0) | 137.0 (100.4, 186.9) | 214.3 (151.7, 302.6) | 235.2 (176.2, 313.8) | 87.2 (38.9, 195.8) | 119.9 (77.9, 184.6) | 191.3 (124.7, 293.5) | 167.8 (103.2, 272.6) |
| GMFR (95% CI) | 3.7 (2.0, 7.0) | 4.8 (3.3, 6.9) | 7.4 (4.8, 11.3) | 6.2 (4.8, 8.0) | 2.3 (1.3, 4.3) | 4.0 (2.9, 5.5) | 7.7 (5.3, 11.1) | 6.7 (5.0, 9.1) | 2.3 (1.1, 4.5) | 2.3 (1.7, 3.2) | 3.5 (2.3, 5.5) | 5.5 (3.5, 8.8) |
| Seroconversion,^c^ n^d^ (%) [95% CI] | 9 (50.0) [26.0, 74.0] | 31 (60.8) [46.1, 74.2] | 37 (71.2) [56.9, 82.9] | 43 (78.2) [65.0, 88.2] | 7 (35.0) [15.4, 59.2] | 29 (51.8) [38.0, 65.3] | 33 (64.7) [50.1, 77.6] | 43 (79.6) [66.5, 89.4] | 4 (33.3) [9.9, 65.1] | 10 (33.3) [17.3, 52.8] | 18 (58.1) [39.1, 75.5] | 20 (69.0) [49.2, 84.7] |
| **Day 181** |  |  |  |  |  |  |  |  |  |  |  |  |
| n^b^ | 18 | 47 | 49 | 54 | 20 | 55 | 49 | 53 | 12 | 29 | 31 | 29 |
| GMT (95% CI) | 100.9 (54.0, 188.5) | 177.4 (124.2, 253.3) | 206.4 (142.4, 299.0) | 232.1 (171.5, 314.1) | 75.9 (40.5, 142.4) | 99.79 (73.8, 135.0) | 132.2 (94.4, 185.1) | 151.9 (113.4, 203.4) | 69.2 (34.0, 141.1) | 93.5 (58.1, 150.3) | 132.3 (88.3, 198.3) | 95.70 (61.8, 148.3) |
| GMFR (95% CI) | 2.4 (1.1, 5.0) | 3.2 (2.2, 4.5) | 5.2 (3.4, 8.1) | 3.9 (3.0, 5.1) | 1.6 (0.5, 2.7) | 2.9 (2.1, 4.0) | 4.8 (3.4, 6.8) | 4.4 (3.2, 5.9) | 1.8 (1.0, 3.3) | 1.8 (1.2, 2.6) | 2.4 (1.6, 3.7) | 3.2 (2.1, 4.8) |
| Seroconversion,^c^ n^d^ (%) [95% CI] | 5 (27.8) [9.7, 53.5] | 23 (48.9) [34.1, 63.9] | 29 (59.2) [44.2, 73.0] | 34 (63.0) [48.7, 75.7] | 3 (15.0) [3.2, 37.9) | 22 (40.0) [27.0, 54.1] | 26 (53.1) [38.3, 67.5] | 33 (62.3) [47.9, 75.2] | 4 (33.3) [9.9, 65.1] | 7 (24.1) [10.3, 43.5) | 13 (41.9) [24.6, 60.9] | 18 (62.1) [42.3, 79.3] |
| **Influenza A/H3N2 Antibodies by HAI Assay** | | | | | | | | | | | | |
| **Baseline  (Day 1)** |  |  |  |  |  |  |  |  |  |  |  |  |
| n^a^ | 20 | 55 | 53 | 57 | 20 | 58 | 54 | 54 | 12 | 31 | 31 | 30 |
| GMT (95% CI) | 22.6 (13.5, 37.6) | 33.9 (24.7, 46.7) | 26.5 (19.0, 36.9) | 46.0 (35.8, 59.1) | 28.7 (15.8, 52.3) | 21.1 (15.0, 29.6) | 18.8 (13.4, 26.2) | 17.8 (13.5, 23.4) | 69.2 (18.9, 253.9) | 28.9 (19.5, 42.9) | 33.5 (20.1, 55.7) | 21.9 (15.0, 32.1) |
| **Day 29** |  |  |  |  |  |  |  |  |  |  |  |  |
| n^b^ | 20 | 55 | 53 | 57 | 20 | 58 | 54 | 54 | 12 | 31 | 31 | 30 |
| GMT (95% CI) | 113.1 (78.0, 164.1) | 255.0 (185.3,350.9) | 279.0 (203.1, 383.3) | 400.8 (315.7, 508.8) | 84.3 (55.5, 128.2) | 144.6 (103.7, 201.5) | 163.1 (114.3, 232.8) | 170.5 (116.9, 248.8) | 116.5 (47.0, 288.7) | 111.9 (69.2, 181.0) | 158.3 (97.5, 257.1) | 160.0 (102.0, 250.8) |
| GMFR (95% CI) | 5.0 (2.9, 8.6) | 7.5 (5.4, 10.5) | 10.5 (7.6, 14.6) | 8.7 (6.7, 11.3) | 2.9 (1.8, 4.8) | 6.9 (4.5, 10.3) | 8.7 (6.1, 12.5) | 9.6 (6.7, 13.7) | 1.7 (0.6, 5.1) | 3.9 (2.7, 5.7) | 4.7 (3.3, 6.9) | 7.3 (5.0, 10.6) |
| Seroconversion,^c^ n^d^ (%) [95% CI] | 10 (50.0) [27.2, 72.8] | 42 (76.4) [63.0, 86.8] | 43 (81.1) [68.0, 90.6] | 47 (82.5) [70.1, 91.3] | 8 (40.0) [19.1, 64.0] | 41 (70.7) [57.3, 81.9] | 38 (70.4) [56.4, 82.0] | 42 (77.8) [64.6, 88.0] | 3 (25.0) [5.5, 57.2 | 12 (38.7) [21.9, 57.8] | 18 (58.1) [39.1, 75.5] | 22 (73.2) [54.1, 87.7] |
| **Day 91** |  |  |  |  |  |  |  |  |  |  |  |  |
| n^b^ | 18 | 51 | 52 | 55 | 20 | 56 | 51 | 54 | 12 | 30 | 31 | 29 |
| GMT (95% CI) | 124.7 (78.7, 197.6) | 204.3 (149.2, 279.9) | 189.0 (133.3, 268.1) | 271.7 (205.6, 359.0) | 77.3 (47.0, 126.9) | 117.4 (84.9, 162.4) | 141.6 (101.0, 198.6) | 124.5 (85.7, 180.9) | 103.7 (45.5, 236.6) | 109.3 (66.6, 179.4) | 144.7 (89.0, 235.2) | 126.0 (72.2, 219.7) |
| GMFR (95% CI) | 6.0 (3.2, 11.3) | 6.4 (4.6, 8.7) | 7.3 (5.3, 10.1) | 6.0 (4.7, 7.7) | 2.7 (1.5, 4.9) | 5.4 (3.6, 8.1) | 8.1 (5.7, 11.4) | 7.0 (5.0, 9.7) | 1.5 (0.6, 4.0) | 3.7 (2.5, 5.5) | 4.3 (3.0, 6.3) | 6.0 (3.9, 9.2) |
| Seroconversion,^c^ n^d^ (%) [95% CI] | 11 (61.1) [35.8, 82.7] | 35 (68.8) [54.1, 80.9] | 40 (76.9) [63.2, 87.5] | 40 (72.7) [59.0, 83.9] | 7 (35.0) [15.4, 59.2] | 33 (58.9) [45.0, 71.9] | 35 (68.6) [54.1, 80.9] | 35 (64.8) [50.6, 77.3] | 2 (16.7) [2.1, 48.4] | 10 (33.3) [17.3, 52.8] | 19 (61.3) [42.2, 78.2] | 22 (75.9) [56.5, 89.7] |
| **Day 181** |  |  |  |  |  |  |  |  |  |  |  |  |
| n^b^ | 18 | 47 | 49 | 54 | 20 | 55 | 49 | 53 | 12 | 29 | 31 | 29 |
| GMT (95% CI) | 88.1 (64.9, 119.7) | 152.0 (112.4, 205.6) | 135.0 (92.5, 197.0) | 171.7 (129.7, 227.2) | 55.5 (34.9, 88.3) | 81.5 (58.5, 113.6) | 89.0 (63.5, 124.6) | 86.5 (60.7, 123.3) | 97.9 (37.5, 255.6) | 78.2 (47.3, 129.3) | 91.5 (57.5, 145.7) | 80.0 (50.8, 126.0) |
| GMFR (95% CI) | 4.2 (2.5, 7.2) | 4.4 (3.3, 5.9) | 5.4 (3.9, 7.5) | 3.8 (3.1, 4.7) | 1.9 (1.1, 3.3) | 3.8 (2.6, 5.5) | 5.4 (4.0, 7.3) | 4.7 (3.5, 6.5) | 1.4 (0.5, 3.8) | 2.7 (1.8, 4.0) | 2.7 (1.9, 3.9) | 3.8 (2.9, 5.1) |
| Seroconversion,^c^ n^d^ (%) [95% CI] | 9 (50.0) [26.0, 74.0] | 29 (61.7) [46.4, 75.5] | 30 (61.2) [46.2, 74.8] | 29 (53.7) [39.6, 67.4] | 4 (20.0) [5.7, 43.7] | 24 (43.6) [30.3, 57.7] | 25 (51.0) [36.3, 65.6] | 26 (49.1) [35.1, 63.2] | 2 (16.7) [2.1, 48.4] | 8 (27.6) [12.7, 47.2] | 9 (29.0) [14.2, 48.1] | 15 (55.2) [35.7, 73.6] |
| **Influenza B/Victoria Lineage Antibodies by HAI Assay** | | | | | | | | | | | | |
| **Baseline  (Day 1)** |  |  |  |  |  |  |  |  |  |  |  |  |
| n^a^ | 20 | 55 | 53 | 56 | 20 | 58 | 54 | 54 | 12 | 31 | 31 | 30 |
| GMT (95% CI) | 33.7 (18.8, 60.1) | 44.8 (32.8, 61.3) | 30.4 (21.2, 61.3) | 36.9 (27.5, 49.5) | 68.4 (38.2, 122.5) | 51.4 (38.6, 68.4) | 55.1 (40.5, 75.0) | 53.0 (40.3, 69.7) | 50.4 (20.3, 125.5) | 51.7 (36.9, 72.5) | 59.8 (34.9, 102.5) | 60.6 (38.9, 94.5) |
| **Day 29** |  |  |  |  |  |  |  |  |  |  |  |  |
| n^b^ | 20 | 55 | 53 | 57 | 20 | 58 | 54 | 54 | 12 | 31 | 31 | 30 |
| GMT (95% CI) | 160.1 (91.9, 278.6) | 99.7 (78.3, 127.0) | 119.1 (93.2, 152.2) | 117.3 (90.7, 151.6) | 183.9 (123.5, 273.9) | 131.1 (100.3, 171.2) | 181.9 (140.5, 235.6) | 149.1 (117.8, 188.7) | 127.1 (47.0, 343.6) | 97.9 (72.4, 132.4) | 104.6 (61.9, 176.9) | 128.6 (80.9, 204.5) |
| GMFR (95% CI) | 4.8 (2.0, 11.1) | 2.2 (1.8, 2.8) | 3.9 (3.0, 5.2) | 3.2 (2.7, 3.9) | 2.7 (1.6, 4.5) | 2.6 (2.1, 3.1) | 3.3 (2.5, 4.3) | 2.8 (2.3, 3.5) | 2.5 (0.8, 8.0) | 1.9 (1.5, 2.4) | 1.8 (1.4, 2.1) | 2.1 (1.7, 2.7) |
| Seroconversion,^c^ n^d^ (%) [95% CI] | 8 (40.0) [19.1, 64.0] | 10 (18.2) [9.1, 30.9] | 21 (39.6) [26.5, 54.0] | 25 (44.6) [31.3, 58.5] | 6 (30.0) [11.9, 54.3] | 18 (31.0) [19.5, 44.5] | 21 (38.9) [25.9, 53.1] | 21 (38.9) [25.9, 53.1] | 3 (25.0) [5.5, 57.2] | 5 (16.1) [5.5, 33.7] | 4 (12.9) [3.6, 29.8] | 6 (20.0) [7.7, 38.6] |
| **Day 91** |  |  |  |  |  |  |  |  |  |  |  |  |
| n^b^ | 18 | 51 | 52 | 55 | 20 | 56 | 51 | 54 | 12 | 30 | 31 | 29 |
| GMT (95% CI) | 50.4 (31.7, 80.0) | 39.4 (30.3, 51.4) | 34.5 (25.1, 47.4) | 39.0 (29.7, 51.3) | 70.9 (45.6, 110.2) | 51.6 (38.2, 69.5) | 58.9 (44.7, 77.7) | 48.8 (39.5, 60.3) | 69.1 (28.0, 170.7) | 42.4 (30.1, 59.8) | 47.8 (30.9, 74.0) | 59.3 (38.7, 90.9) |
| GMFR (95% CI) | 1.7 (0.9, 3.1) | 0.9 (0.7, 1.1) | 1.2 (0.9, 1.6) | 1.1 (0.9, 1.3) | 1.0 (0.6, 1.9) | 1.0 (0.8, 1.2) | 1.1 (0.8, 1.5) | 0.9 (0.7, 1.2) | 1.4 (0.5, 3.7) | 0.8 (0.6, 1.1) | 0.8 (0.6, 1.0) | 1.0 (0.8, 1.4) |
| Seroconversion,^c^ n^d^ (%) [95% CI] | 5 (27.8) [9.7, 53.5] | 4 (7.8) [2.2, 18.9] | 5 (9.6) [3.2, 21.0] | 2 (3.7) [0.5, 12.8] | 3 (15.0) [3.2, 37.9] | 4 (7.1) [2.0, 17.3] | 5 (9.8) [3.3, 21.4] | 4 (7.4) [2.1,17.9] | 2 (16.7) [2.1, 48.4] | 1 (3.3) [0.1, 17.2] | 0 [0.0, 11.2] | 1 (3.4) [0.1, 17.8] |
| **Day 181** |  |  |  |  |  |  |  |  |  |  |  |  |
| n^b^ | 18 | 47 | 49 | 54 | 20 | 55 | 49 | 53 | 12 | 29 | 31 | 29 |
| GMT (95% CI) | 42.3 (24.4, 73.4) | 35.2 (26.7, 46.5) | 28.1 (20.8, 38.0) | 31.1 (23.5, 41.2) | 55.6 (36.2, 85.3) | 44.8 (33.6, 59.7) | 50.9 (37.7, 68.6) | 39.0 (30.5, 49.8) | 48.9 (22.3, 107.0) | 35.0 (25.3, 48.5) | 40.4 (26.1, 62.7) | 46.7 (29.7, 73.5) |
| GMFR (95% CI) | 1.4 (0.7, 2.8) | 0.7 (0.6, 0.9) | 0.9 (0.7, 1.3) | 0.9 (0.7, 1.1) | 0.8 (0.5, 1.4) | 0.9 (0.7, 1.0) | 1.0 (0.8, 1.3) | 0.7 (0.6, 0.9) | 1.0 (0.5, 2.1) | 0.7 (0.5, 0.9) | 0.7 (0.5, 0.9) | 0.8 (0.7, 1.0) |
| Seroconversion,^c^ n^d^ (%) [95% CI] | 4 (22.2) [6.4, 47.6] | 2 (4.3) [0.5, 14.5] | 3 (6.1) [1.3, 16.9] | 1 (1.9) [0.1, 10.1] | 1 (5.0) [0.1, 24.9] | 2 (3.4) [0.4, 12.5] | 5 (10.2) [3.4, 22.2] | 2 (3.8) [0.5, 13.0] | 2 (16.7) [2.1, 48.4] | 1 (3.4) [0.1, 17.8] | 0 [0.0, 11.2] | 0 [0.0, 11.9] |
| **Influenza B/Yamagata Lineage Antibodies by HAI Assay** | | | | | | | | | | | | |
| **Baseline  (Day 1)** |  |  |  |  |  |  |  |  |  |  |  |  |
| n^a^ | 20 | 55 | 53 | 57 | 20 | 58 | 54 | 54 | 12 | 31 | 31 | 30 |
| GMT (95% CI) | 95.1 (52.0, 173.9) | 89.0 (65.0, 121.9) | 60.0 (41.4, 86.9) | 79.1 (59.4, 105.2) | 61.6 (36.4, 104.3) | 64.5 (46.3, 89.8) | 59.1 (41.0, 85.4) | 65.1 (48.3, 87.8) | 42.3 (18.3, 98.0) | 61.2 (42.0, 89.1) | 100.0 (55.9, 178.8) | 54.1 (35.0, 83.5) |
| **Day 29** |  |  |  |  |  |  |  |  |  |  |  |  |
| n^b^ | 20 | 55 | 53 | 57 | 20 | 58 | 54 | 54 | 12 | 31 | 31 | 30 |
| GMT (95% CI) | 387.0 (239.1, 626.3) | 314.0 (245.7, 401.4) | 309.7 (223.8, 428.5) | 313.5 (239.2, 410.9) | 165.6 (102.3, 268.2) | 210.7 (159.7, 277.9) | 226.3 (174.0, 294.4) | 255.7 (201.8, 324.0 | 100.9 (63.5, 160.2) | 191.4 (137.0, 267.4) | 204.6 (131.2, 319.0) | 151.0 (94.2, 242.2) |
| GMFR (95% CI) | 4.1 (2.1, 8.0) | 3.5 (2.7, 4.7) | 5.2 (3.6, 7.4) | 4.0 (3.0, 5.2) | 2.7 (1.7, 4.3) | 3.3 (2.6, 4.1) | 3.8 (3.0, 4.9) | 3.9 (3.2, 4.9) | 2.4 (1.2, 4.7) | 3.1 (2.1, 4.6) | 2.1 (1.5, 2.8) | 2.8 (2.0, 3.9) |
| Seroconversion,^c^ n^d^ (%) [95% CI] | 8 (40.0) [19.1, 64.0] | 22 (40.0) [27.0, 54.1] | 30 (56.6) [42.3, 70.2] | 35 (61.4) [47.6, 74.0] | 5 (25.0) [8.7, 49.1] | 27 (46.6) [33.3, 60.1] | 29 (53.7) [39.6, 67.4] | 27 (50.0) [36.1, 63.9] | 4 (33.3) [9.9, 65.1] | 11 (35.5) [19.2, 54.6] | 7 (22.6) [9.6, 41.1] | 11 (36.7) [19.9, 56.1] |
| **Day 91** |  |  |  |  |  |  |  |  |  |  |  |  |
| n^b^ | 18 | 51 | 52 | 55 | 20 | 56 | 51 | 54 | 12 | 30 | 31 | 29 |
| GMT (95% CI) | 137.3 (89.3, 211.2) | 111.7 (89.0, 140.0) | 113.9 (82.7, 156.8) | 126.6 (99.6, 161.0) | 80.0 (49.0, 130.7) | 89.4 (67.8, 118.0) | 94.8 (74.6, 120.5) | 81.0 (62.1, 105.7) | 61.7 (35.9, 105.9) | 70.5 (53.0, 93.8) | 85.6 (58.2, 126.0) | 71.8 (46.0, 112.2) |
| GMFR (95% CI) | 1.7 (0.9, 3.1) | 1.3 (0.9, 1.8) | 2.0 (1.4, 2.8) | 1.6 (1.2, 2.1) | 1.3 (0.8, 2.2) | 1.4 (1.1, 1.8) | 1.7 (1.3, 2.3) | 1.2 (1.0, 1.6) | 1.5 (0.8, 2.6) | 1.1 (0.9, 1.4) | 0.9 (0.6, 1.2) | 1.4 (0.9, 2.0) |
| Seroconversion,^c^ n^d^ (%) [95% CI] | 4 (22.2) [6.4, 47.6] | 9 (17.6) [8.4, 30.9] | 13 (25.0) [14.0, 39.0.] | 9 (16.4) [7.9, 28.8] | 3 (15.0) [3.2, 37.9] | 7 (12.5) [5.2, 24.1] | 12 (23.5) [12.8, 37.5] | 8 (14.8) [6.6, 27.1] | 2 (16.7) [2.1, 48.4] | 1 (3.3) [0.1, 17.2] | 2 (6.5) [0.8, 21.4] | 5 (17.2) [5.9, 35.8] |
| **Day 181** |  |  |  |  |  |  |  |  |  |  |  |  |
| n^b^ | 18 | 47 | 49 | 54 | 20 | 55 | 49 | 53 | 12 | 29 | 31 | 29 |
| GMT (95% CI) | 115.4 (79.3, 168.0) | 90.0 (71.1, 114.0) | 92.8 (66.3, 130.0) | 93.4 (72.1, 121.0) | 54.6 (36.5, 81.8) | 74.6 (56.2, 99.0) | 66.6 (50.3, 88.0) | 63.6 (48.7, 83.0) | 47.6 (27.0, 84.0) | 51.4 (37.1, 71.2) | 65.3 (43.5, 98.1) | 54.5 (37.5, 79.3) |
| GMFR (95% CI) | 1.4 (0.8, 2.4) | 1.0 (0.8, 1.4) | 1.6 (1.1, 2.4) | 1.2 (0.9, 1.5) | 0.9 (0.6, 1.4) | 1.1 (0.9, 1.4) | 1.3 (1.0, 1.7) | 1.0 (0.8, 1.3) | 1.1 (0.7, 1.9) | 0.8 (0.6, 1.0) | 0.7 (0.5, 0.9) | 1.1 (0.8, 1.5) |
| Seroconversion,^c^ n^d^ (%) [95% CI] | 3 (16.7) [3.6, 41.4] | 5 (10.6) [3.6, 23.1) | 12 (24.5) [13.3, 38.9] | 4 (7.4) [2.1, 17.9] | 1 (5.0) [0.1, 24.9] | 5 (9.1) [3.0, 20.0] | 4 (8.2) [2.3, 19.6] | 5 (9.4) [3.1, 20.7] | 0 [0.0, 26.5] | 1 (3.4) [0.1, 17.8] | 1 (3.2) [0.1, 16.7] | 2 (6.9) [0.9, 22.8] |

The per-protocol population comprised all randomly assigned participants who received the vaccination and who did not have influenza infection at baseline through Day 29 and had no major protocol deviations that could impact the immune response.

CI, confidence interval; GMFR, geometric mean fold rise; GMT, geometric mean titer; HA, hemagglutinin; HAI, hemagglutination inhibition; mRNA, messenger RNA.

^a^Number of subjects with non-missing baseline (Day 1) anti-HA antibody data.

^b^Number of subjects with non-missing anti-HA antibody data at the corresponding visit.

^c^Seroconversion at a subject level is defined as a post-baseline titer ≥1:40 if baseline is <1:10 or a 4-fold or greater rise if baseline is ≥1:10 in anti-HA antibodies.

^d^Number of subjects meeting the criterion at the corresponding visit. Percentage is based on the number of subjects with non-missing baseline (Day 1) anti-HA antibody (n^b^ rows).

**Table S5.** Summary of Influenza Antibody Responses by HAI at Day 29 by Age Group in Study Part 3 (Per-Protocol Population)

|  | **Part 3** | | | | | | | | | | | | | | | | |
| --- | --- | --- | --- | --- | --- | --- | --- | --- | --- | --- | --- | --- | --- | --- | --- | --- | --- |
|  | **18-49 Years** | | | | | | | | **≥50 Years** | | | | | | | | |
|  | **Afluria**  **(n=32)** | **6.25 µg mRNA-1010**  **(n=34)** | | **12.5 µg mRNA-1010 (n=32)** | | **25 µg mRNA-1010 (n=31)** | | | **Afluria**  **(n=16)** | | | **6.25 µg mRNA-1010**  **(n=15)** | | | **12.5 µg mRNA-1010 (n=15)** | | **25 µg mRNA-1010 (n=15)** |
| **Influenza A H1N1 Antibodies by HAI Assay** | | | | | | | | | | | | | | | | | |
| **Baseline  (Day 1)** |  |  | |  | |  | | |  | | |  | | |  | |  |
| n^a^ | 32 | 34 | | 32 | | 31 | | | 16 | | | 15 | | | 15 | | 15 |
| GMT (95% CI) | 42.2 (26.9, 66.2) | 40.8 (27.9, 59.7) | | 56.6 (34.9, 91.7) | | 44.2 (25.7, 76.1) | | | 59.1  (30.0, 116.3) | | | 43.9 (24.5, 78.6) | | | 43.9 (21.6, 89.1) | | 33.3 (17.8, 62.3) |
| **Day 29** |  |  | |  | |  | | |  | | |  | | |  | |  |
| n^b^ | 32 | 34 | | 32 | | 31 | | | 16 | | | 15 | | | 15 | | 15 |
| GMT (95% CI) | 323.4  (194.9, 536.7) | 291.9  (193.5, 440.2) | | 482.8  (312.5, 745.7) | | 715.7  (505.4, 1013.6) | | | 226.4  (103.4, 495.4) | | | 179.4  (101.9, 316.0) | | | 221.1  (119.2, 410.0) | | 248.1  (94.1, 650.6) |
| GMFR (95% CI) | 7.7 (4.2, 13.9) | 7.2 (4.6, 11.2) | | 8.5 (5.0, 14.5) | | 16.2 (9.7, 27.1) | | | 3.8 (1.8, 8.0) | | | 4.1 (2.3, 7.4) | | | 5.0 (3.1, 8.2) | | 7.5 (2.9, 19.4) |
| Seroconversion,^c^ n^d^ (%) [95% CI] | 17 (53.1)  [34.7, 70.9] | 21 (61.8)  [43.6, 77.8] | | 21 (65.6)  [46.8, 81.4] | | 26 (83.9)  [66.3, 94.6] | | | 7 (43.8)  [19.8, 70.1] | | | 8 (53.3)  [26.6, 78.7] | | | 11 (73.3)  [44.9, 92.2] | | 8 (53.3)  [26.6, 78.7] |
| **Influenza A H3N2 Antibodies by HAI Assay** | | | | | | | | | | | | | | | | | |
| **Baseline  (Day 1)** |  |  | |  | |  | | |  | | |  | | |  | |  |
| n^a^ | 32 | 34 | | 32 | | 31 | | 16 | | | 15 | | | 15 | | 15 | |
| GMT (95% CI) | 42.7 (26.6, 68.5) | 46.6 (29.5, 73.7) | | 42.2 (27.1, 65.9) | | 39.1 (26.2, 58.5) | | 16.1 (9.9, 26.0) | | | 33.3 (14.6, 75.5) | | | 24.0 (13.0, 44.2) | | 27.6 (12.6, 60.4) | |
| **Day 29** |  |  | |  | |  | |  | | |  | | |  | |  | |
| n^b^ | 32 | 34 | | 32 | | 31 | | 16 | | | 15 | | | 15 | | 15 | |
| GMT (95% CI) | 186.3  (118.5, 293.1) | 200.2 (125.3, 320.0) | | 313.0  (208.4, 470.1) | | 357.9  (283.0, 538.1) | | 45.5 (21.8, 95.0) | | | 167.5  (102.0, 275.3) | | | 127.1  (62.3, 259.3) | | 266.0  (149.7, 472.8) | |
| GMFR (95% CI) | 4.4 (2.6, 7.5) | 4.3 (2.7, 6.7) | | 7.4 (4.7, 11.6) | | 9.2 (5.7, 14.7) | | 2.8 (1.7, 4.8) | | | 5.0 (2.6, 9.7) | | | 5.3 (2.5, 11.2) | | 9.6 (3.7, 24.8) | |
| Seroconversion,^c^ n^d^ (%) [95% CI] | 15 (46.9)  [29.1, 65.3] | 15 (44.1)  [27.2, 62.1] | | 22 (68.8)  [50.0, 83.9) | | 25 (80.6)  [62.5, 92.6] | | 6 (37.5)  [15.2, 64.7] | | | 7 (46.7)  [21.3, 73.4] | | | 9 (60.0)  [32.3, 83.7] | | 12 (80.0)  [51.9, 95.7] | |
| **Influenza B/Victoria Lineage Antibodies by HAI Assay** | | | | | | | | | | | | | | | | | |
| **Baseline  (Day 1)** |  |  | |  | |  | |  | | |  | | |  | |  | |
| n^a^ | 32 | 34 | | 32 | | 31 | | 16 | | | 15 | | | 15 | | 15 | |
| GMT (95% CI) | 47.6 (31.2, 72.7) | 28.0 (19.2, 40.8) | | 37.9 (23.9, 60.1) | | 28.3 (18.0, 44.4) | | 34.4 (18.3, 64.4) | | | 41.0 (20.6, 81.4) | | | 45.9 (26.0, 81.3) | | 43.9 (20.1, 96.0) | |
| **Day 29** |  |  | |  | |  | |  | | |  | | |  | |  | |
| n^b^ | 32 | 34 | | 31 | | 31 | | 16 | | | 15 | | | 15 | | 15 | |
| GMT (95% CI) | 186.2 (140.0, 247.8) | 51.1 (35.6, 73.3) | | 77.4 (60.2, 99.4) | | 73.1  (50.2, 106.5) | | 134.5  (85.6, 211.5) | | | 57.9  (29.4, 114.1) | | | 71.3  (40.2, 126.6) | | 98.4  (47.8, 202.5) | |
| GMFR (95% CI) | 3.9 (2.4, 6.4) | 1.8 (1.3, 2.6) | | 2.0 (1.4, 3.0) | | 2.6 (1.7, 4.0) | | 3.9 (2.0, 7.7) | | | 1.4 (1.1, 1.8) | | | 1.6 (1.1, 2.2) | | 2.2 (1.3, 3.9) | |
| Seroconversion,^c^ n^d^ (%) [95% CI] | 14 (43.8)  [26.4, 62.3] | 5 (14.7)  [5.0, 31.1] | | 8 (25.8)  [11.9, 44.6] | | 10 (32.3)  [16.7. 51.4] | | 9 (56.3)  [29.9, 80.3] | | | 0  [0.0, 21.8] | | | 2 (13.3)  [1.7, 40.5] | | 3 (20.0)  [4.3, 48.1] | |
| **Influenza B/Yamagata Lineage Antibodies by HAI Assay** | | | | | | | | | | | | | | | | | |
| **Baseline (Day 1)** |  | |  | |  | |  | | |  | | |  | |  | |  |
| n^a^ | 32 | 34 | | 32 | | 31 | | 16 | | | 15 | | | 15 | | 15 | |
| GMT (95% CI) | 75.8  (47.1, 121.9) | 58.3  (39.2, 86.8) | | 86.3  (55.4, 134.4) | | 54.7  (31.7, 94.3) | | 32.2 (22.1, 46.8) | | | 62.0  (32.0, 120.4) | | | 47.1 (29.6, 74.8) | | 36.4 (19.8, 66.9) | |
| **Day 29** |  |  | |  | |  | |  | | |  | | |  | |  | |
| n^b^ | 32 | 34 | | 31 | | 31 | | 16 | | | 15 | | | 15 | | 15 | |
| GMT (95% CI) | 303.1  (205.5, 447.2) | 152.1  (98.3, 235.3) | | 302.7  (218.7, 418.9) | | 195.7  (131.3, 291.7) | | 103.7  (60.4, 178.3) | | | 105.6  (63.8, 174.7) | | | 110.6  (60.4, 202.4) | | 82.0  (44.6, 150.6) | |
| GMFR (95% CI) | 4.0 (2.7, 5.9) | 2.6 (1.7, 4.0) | | 3.4 (2.2, 5,4) | | 3.6 (2.1, 6.0) | | 3.2 (1.7, 6.3) | | | 1.7 (1.2, 2.4) | | | 2.4 (1.6, 3.5) | | 2.3 (1.3, 4.0) | |
| Seroconversion,^c^ n^d^ (%) [95% CI] | 17 (53.1)  [34.7, 70.9] | 10 (29.4)  [15.1, 47.5] | | 14 (45.2)  [27.3, 64.0] | | 13 (41.0)  [24.6, 60.9] | | 6 (37.5)  [15.2, 64.6] | | | 2 (13.3) [1.7, 40.5] | | | 6 (40.0)  [16.3, 67.7] | | 5 (33.3)  [11.8, 61.6] | |

The per-protocol population comprised all randomly assigned participants who received the vaccination and who did not have influenza infection at baseline through Day 29 and had no major protocol deviations that could impact the immune response.

CI, confidence interval; GMFR, geometric mean fold rise; GMT, geometric mean titer; HA, hemagglutinin; HAI, hemagglutination inhibition; mRNA, messenger RNA.

^a^Number of subjects with non-missing baseline (Day 1) anti-HA antibody data.

^b^Number of subjects with non-missing anti-HA antibody data at the corresponding visit.

^c^Seroconversion at a subject level is defined as a post-baseline titer ≥1:40 if baseline is <1:10 or a 4-fold or greater rise if baseline is ≥1:10 in anti-HA antibodies.

^d^Number of subjects meeting the criterion at the corresponding visit. Percentage is based on the number of subjects with non-missing baseline (Day 1) anti-HA antibody (n^b^ rows).

**Supplemental Figures**

**Figure S1.** Overview of the study design.


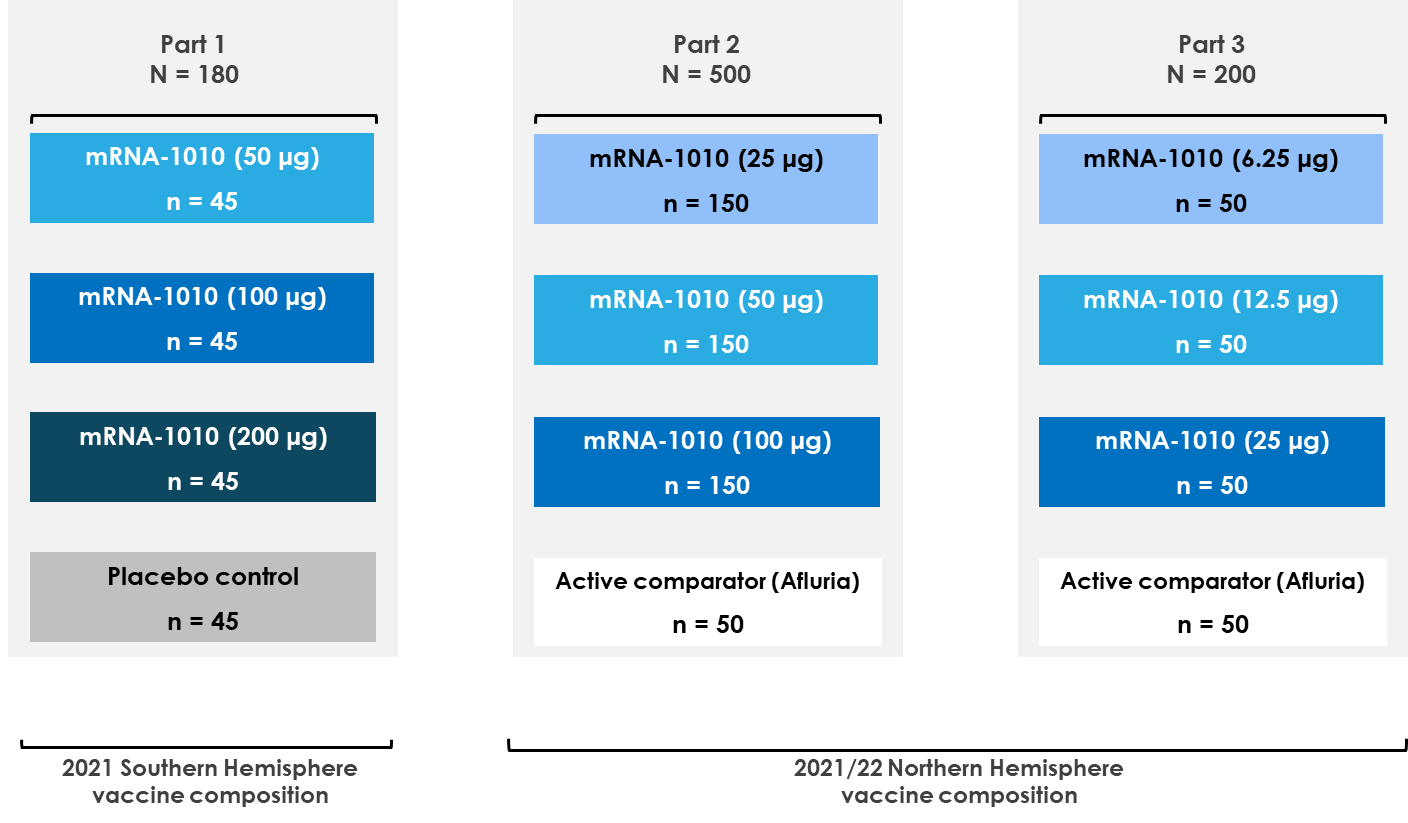


**Figure S2.** Any solicited local and systemic adverse reactions within 7 days after vaccination by participant age group in all study parts combined. Participants aged 18-49 years and ≥50 years received either placebo, or mRNA-1010 50 μg, 100 μg, or 200 μg in Part 1; participants aged 18-49 years, 50-64 years, and ≥65 years received Afluria or mRNA-1010 25 μg, 50 μg, or 100 μg in Part 2; participants received Afluria or mRNA-1010 6.25 μg, 12.5 μg, or 25 μg in Part 3. Percentages of (A) local or (B) systemic adverse reactions are based on overall participants in the solicited safety population for aged 18-49 years and ≥50 years. Number of participants in the placebo group were 20 (18-49 years) and 24 (≥50 years); number of participants in the Afluria group were 56 (18-49 years) and 48 (≥50 years); number of participants in the mRNA-1010 groups were (18-49 years and ≥50 years, respectively) 35 and 15 (6.25 μg), 34 and 16 (12.5 μg), 94 and 104 (25 μg), 80 and 111 (50 μg), 79 and 112 (100 μg), and 23 and 21 (200 μg).


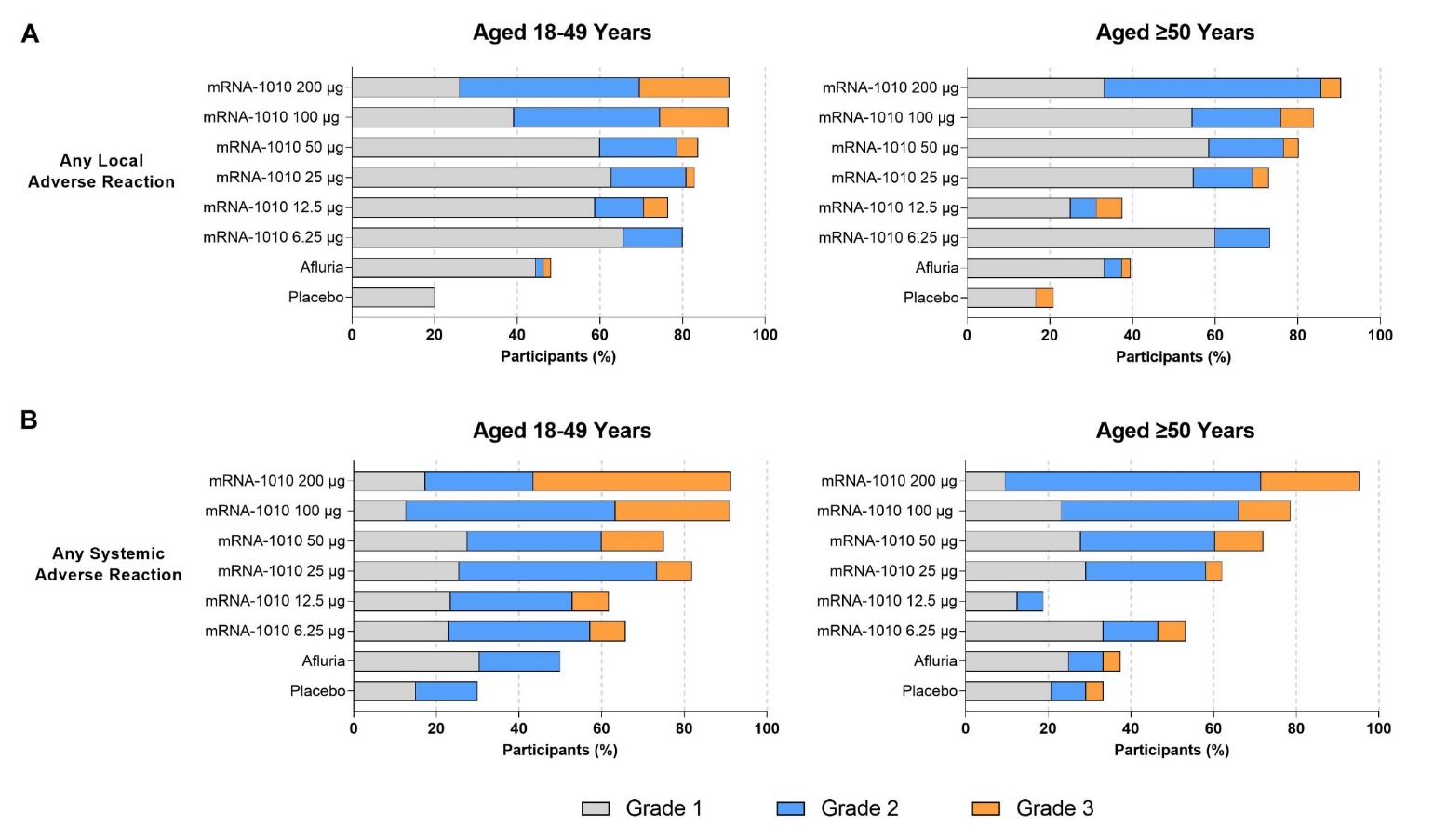


Figure S3. Summary of solicited local and systemic adverse reactions within 7 days after vaccination by participant age group in all study parts combined. Participants aged 18-49 years and ≥50 years received either placebo, or mRNA-1010 50 μg, 100 μg, or 200 μg in Part 1; participants aged 18-49 years, 50-64 years, and ≥65 years received Afluria or mRNA-1010 25 μg, 50 μg, or 100 μg in Part 2; participants received Afluria or mRNA-1010 6.25 μg, 12.5 μg, or 25 μg in Part 3. Percentages of (A) local and (B) systemic adverse reactions are based on overall participants in the solicited safety population. Number of participants in the placebo group were 20 (18-49 years) and 24 (≥50 years); number of participants in the Afluria group were 56 (18-49 years) and 48 (≥50 years); number of participants in the mRNA-1010 groups were (18-49 years and ≥50 years, respectively) 35 and 15 (6.25 μg), 34 and 16 (12.5 μg), 94 and 104 (25 μg), 80 and 111 (50 μg), 79 and 112 (100 μg), and 23 and 21 (200 μg).


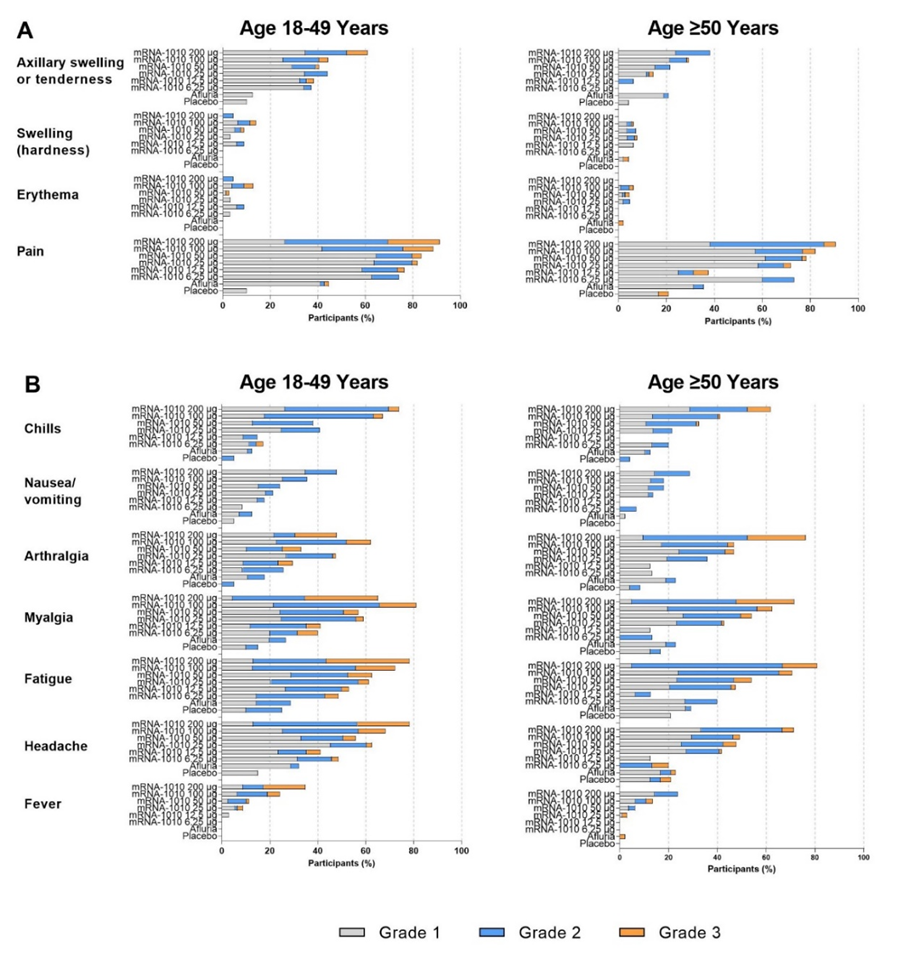


**Figure S4.** Persistence of HAI titers through 6 months after mRNA-1010 vaccination among the different age groups in Part 2. GMTs against vaccine-matched seasonal influenza strains at Day 1 (baseline), Day 29, Day 91, and Day 181 by age groups (A) 18 to 49 years, (B) 50 to 64 years, (C) ≥65 years. Horizontal dotted line indicates 1:40 titer associated with a 50% reduction in risk of infection. Numbers of participants (A) 18-49 years: Afluria 20, mRNA-1010 55 (25 µg), 53 (50 µg), and 57 (100 µg); (B) 50-64 years: Afluria 20, mRNA-1010 58 (25 µg), 54 (50 µg), and 54 (100 µg); (C) ≥65 years: Afluria 12; mRNA-1010 31 (25 µg), 31 (50 µg), and 30 (100 µg). Numbers of participants derived from the per-protocol population. Error bars represent 95% CI.

CI, confidence interval; GMT, geometric mean titer; HAI, hemagglutination inhibition.


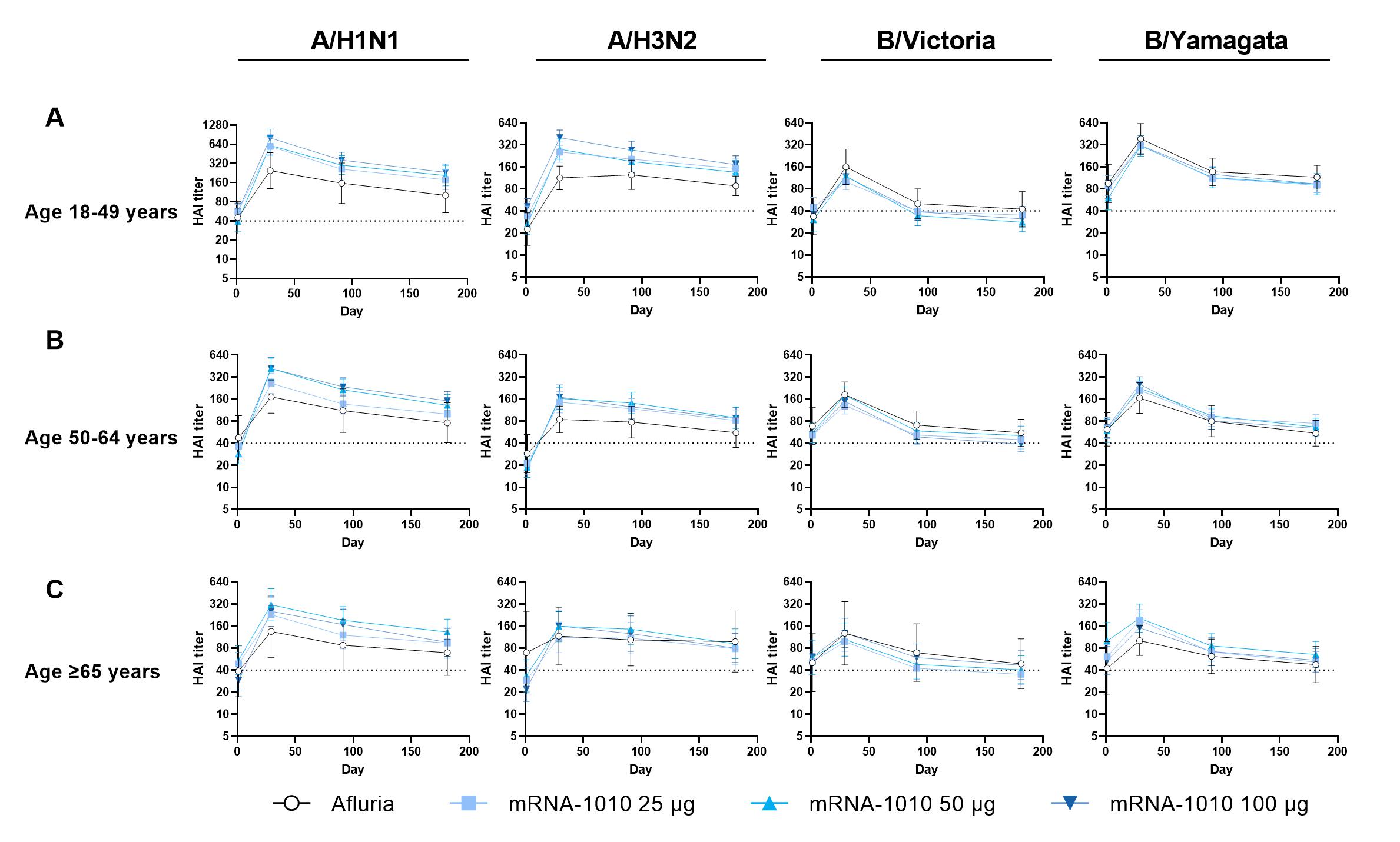


**Figure** **S5.** HAI GMTs from baseline to Day 29 after vaccination with mRNA-1010 against vaccine-matched and vaccine-heterologous A/H3N2 strains in Part 2. GMTs with associated 95% CIs at Day 1 (baseline) and Day 29 against vaccine-matched H3N2 (A/Cambodia/e0826360/2020) and vaccine-heterologous H3N2 strains (A/Newcastle/01/2021, A/Delaware/39/2019, and A/Darwin/11/2021) for mRNA-1010 50 µg and Afluria recipients in Part 2. Numbers of participants for the vaccine-matched strain were 52 (Afluria) and 138 (mRNA-1010 50 µg); for vaccine-heterologous strains, 52 (Afluria) and 139 (mRNA-1010 50 µg). Numbers of participants for the vaccine-matched strain and vaccine-heterologous strains were derived from the per-protocol population.


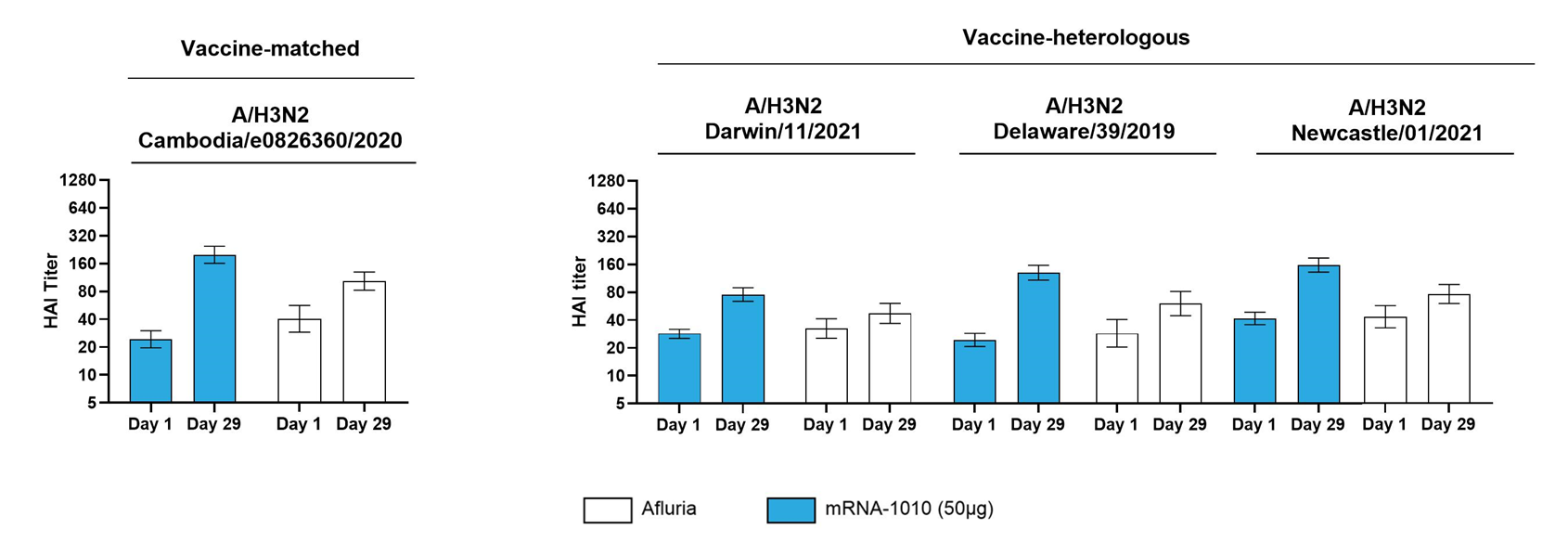


Figure S6. Antigenic breadth of mRNA-1010 against vaccine-heterologous A/H3N2 strains at Day 29 in Part 1. (A) GMFR of titers from baseline to Day 29 with associated 95% CIs among mRNA-1010 50 µg recipients against vaccine-matched A/H3N2 (2021 Southern Hemisphere [A/Hong Kong/45/2019]) and vaccine-heterologous A/H3N2 strains (A/Kansas/14/2017, A/South Australia/34/2019, A/Cambodia/E0826360/2020, and A/Darwin/11/2021). Numbers of participants were 43 (22 in 18-49 age group; 21 in >50 age group). Numbers of participants were derived from the per-protocol population. (B) Phylogenetic tree demonstrating real-time tracking of A/H3N2 clades adapted from Nextstrain.org [3].


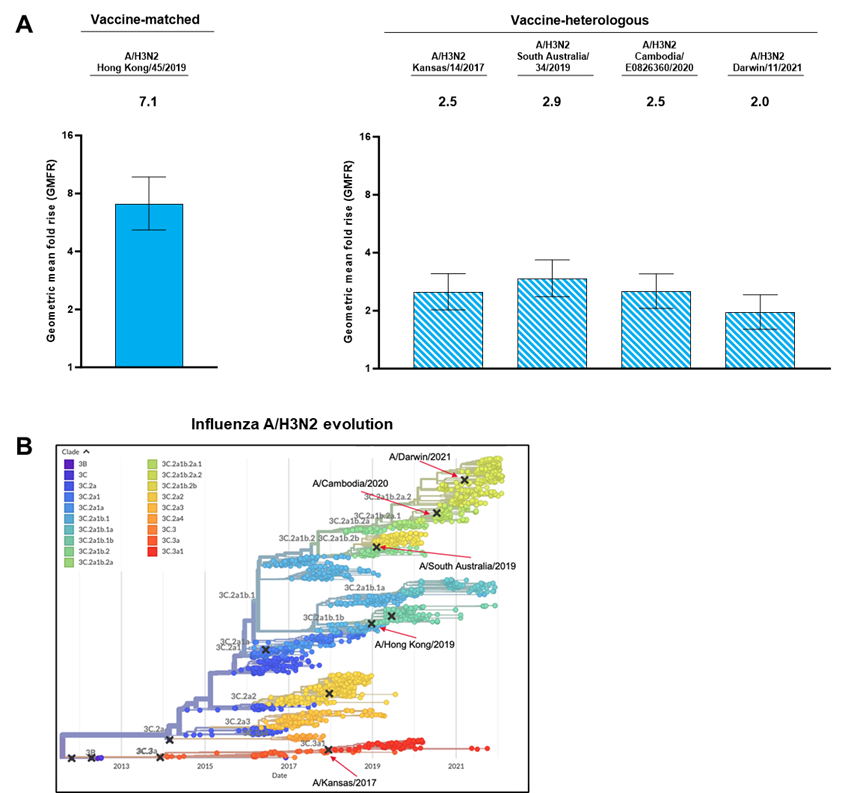


Figure S7. Frequency of CD4+ T-cells minimally expressing IFN-γ and CD40L responses in Part 1. Polyfunctional CD4+ T-cell response in vaccinated groups following ex vivo stimulation with vaccine strain specific peptide pools. T-cell responses were assessed in a subset of Part 1 participants that had adequate aliquots of PBMC at baseline, Day 8, and Day 29. Numbers of participants in the placebo group were 23, and in the mRNA-1010 groups were 19 (50 µg), 28 (100 µg), and 24 (200 µg). Boxes represent the 25th and 75th percentiles; whiskers represent the minimum and maximum values. Symbols represent individual-level data.
IFN, interferon; PBMC, peripheral blood mononuclear cells.

**
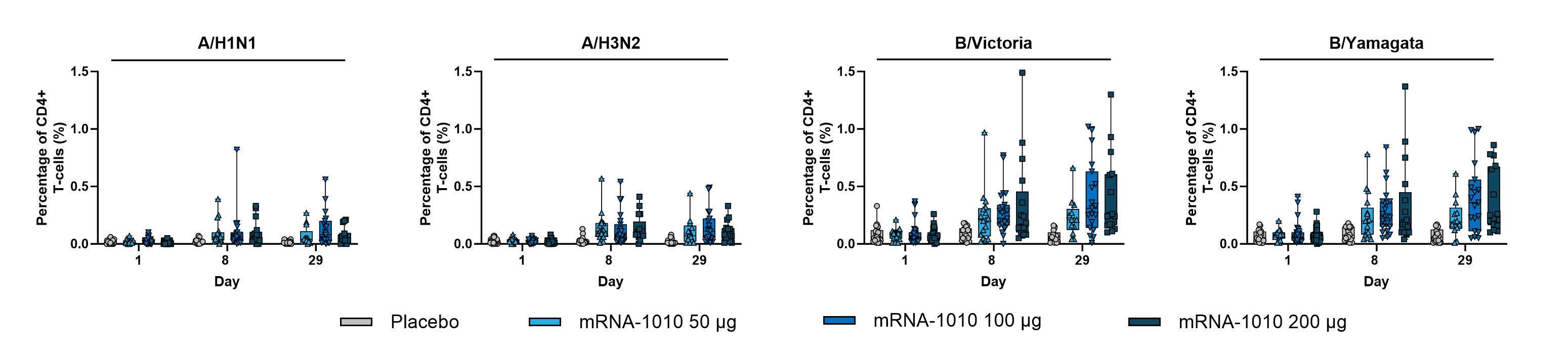
**

**Figure S8.** Frequency of IFN-γ producing CD8+ T-cell responses in Part 1. CD8+ T-cells expressing IFN-γ upon ex vivo stimulation with vaccine strain specific peptide pools. T-cell responses were assessed in a subset of Part 1 participants who had adequate aliquots of PBMC at baseline, Day 8, and Day 29. Numbers of participants in the placebo group were 23, and in the mRNA-1010 groups were 19 (50 µg), 28 (100 µg), and 24 (200 µg). Boxes represent the 25th and 75th percentiles; whiskers represent the minimum and maximum values. Symbols represent individual-level data.

IFN, interferon; PBMC, peripheral blood mononuclear cells.


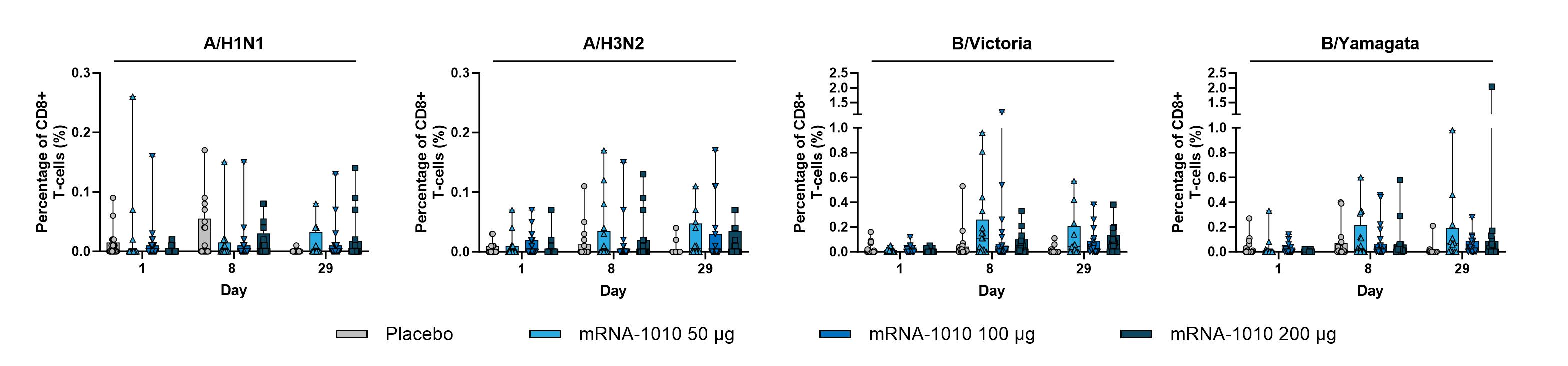


Supplementary References

1. Nachbagauer, R., et al., *A universal influenza virus vaccine candidate confers protection against pandemic H1N1 infection in preclinical ferret studies.* NPJ Vaccines, 2017. **2**: p. 26.

2. Lee, I.T., et al., *Safety and immunogenicity of a phase 1/2 randomized clinical trial of a quadrivalent, mRNA-based seasonal influenza vaccine (mRNA-1010) in healthy adults: interim analysis.* Nat Commun, 2023. **14**(1): p. 3631.

3. Hadfield, J., et al., *Nextstrain: real-time tracking of pathogen evolution.* Bioinformatics, 2018. **34**(23): p. 4121-4123.
